# Supplementary material for: Colloidal pathways of amorphous calcium carbonate formation lead to distinct water environments and conductivity
Source: Nat Commun. 2024 Jan 2;15:80. doi: 10.1038/s41467-023-44381-x (PMC10761707; doi:10.1038/s41467-023-44381-x)
Supplement: Supplementary file 1 — Supplementary Information [file 41467_2023_44381_MOESM1_ESM.pdf]

Supplementary Information for

Colloidal pathways of amorphous calcium carbonate formation lead to distinct water environments and conductivity

Maxim B. Gindele,[a] Sanjay Vinod-Kumar,[b] Johannes Rochau,[a] Daniel Boemke,[a] Eduard Groß,[a] Venkata SubbaRao Redrouthu,[b] Denis Gebauer*[a] and Guinevere Mathies*[b]

[a] Institute of Inorganic Chemistry, Leibniz University Hannover, Callinstr. 9, 30167 Hannover (Germany), E-mail: gebauer@acc.uni-hannover.de

[b] Department of Chemistry, University of Konstanz, Universitätsstr. 10, 78464 Konstanz (Germany), E-mail: guinevere.mathies@uni-konstanz.de

Table of Contents

1. Figures 3

Supplementary Figure 1: Quantitative evaluation of titration experiments 3

Supplementary Figure 2: ^{1}H-^{13}C cross-polarization spectra of PAsp and PAsp-stabilized ACC 4

Supplementary Figure 3: ^{1}H-^{13}C FSLG HETCOR spectrum of PAsp-stablized ACC 5

Supplementary Figure 4: Quantitative analysis of bicarbonate content in PAsp-stabilized ACC 6

Supplementary Figure 5: TGA and DSC analysis of ACC, PAsp and reference samples 7

Supplementary Figure 6: ATR-FTIR spectra of polymer and ACC samples 8

Supplementary Figure 7: TGA-MS analysis of PAsp-stabilized ACC and reference samples 9

Supplementary Figure 8: TGA analysis of polymer and ACC samples 11

Supplementary Figure 9: TGA-IR analysis of PAsp-stabilized ACC and reference samples 12

Supplementary Figure 10: XRD analysis of polymer stabilized ACC after TGA 14

Supplementary Figure 11: Experimental and numerically simulated ^{1}H spectra 15

Supplementary Figure 12: Contribution of PAsp to ^{1}H NMR spectrum of PAsp-stablized ACC 17

Supplementary Figure 13: ^{1}H Background subtraction 18

Supplementary Figure 14: ^{1}H-^{13}C WISE spectrum of PAsp-stablized ACC at 5 kHz spinning frequency 19

Supplementary Figure 15: ^{1}H-^{13}C WISE spectrum of PAsp-stablized ACC at 10 kHz spinning frequency 20

Supplementary Figure 16: ^{1}H MAS NMR spectra of monohydrocalcite and numerical simulations 21
Supplementary Figure 17: Simulated ^{1}H MAS NMR spectra of a water molecule undergoing isotropic motion 22
Supplementary Figure 18: ^{1}H MAS NMR spectra of PAsp-stabilized ACC at 25°C and -25°C 23
Supplementary Figure 19: Effect of 180 degree flips on ^{1}H-^{13}C CP efficiency 24
Supplementary Figure 20: ^{1}H-^{13}C cross-polarization spectra of PAsp-stablized ACC at 2 kHz spinning frequency 25
Supplementary Figure 21: C-AFM analysis of reference samples 26
Supplementary Figure 22: AFM height map of PAsp-stabilized ACC particles 27
Supplementary Figure 23: C-AFM analysis of polymer-free ACC particles 28
Supplementary Figure 24: C-AFM analysis of PAsp--stabilized ACC particles 30
Supplementary Figure 25: Characterization of synthesized Monohydrocalcite 31

2. Calculation of bicarbonate binding 32

2.1. **Calculation of free ion products 32**
2.1.1. Calibration of Ca-ISE 32
2.1.2. Calculation of free ion products 32

2.2. Calculation of microscopic binding parameters of PNC association 32

2.3. Calculation of bicarbonate binding 33
2.3.1. Experimental strategy 33
2.3.2. Determination of bound carbonate via bound Ca^{2+} 34
2.3.3. Determination of "visible" bound carbonate via NaOH addition 36
2.3.4. Determination of amount of bound bicarbonate 37

3. Discussion of titration experiments and additive-controlled mineralization 39

3.1. Basic titration experiments 39

3.2. Characterization of isolated ACC samples 40

4. Determination of single particle conductivity using C-AFM 47

4.1. Experimental strategy 47

4.2. Evaluation of C-AFM data 49

Supplementary References 51

1. Figures

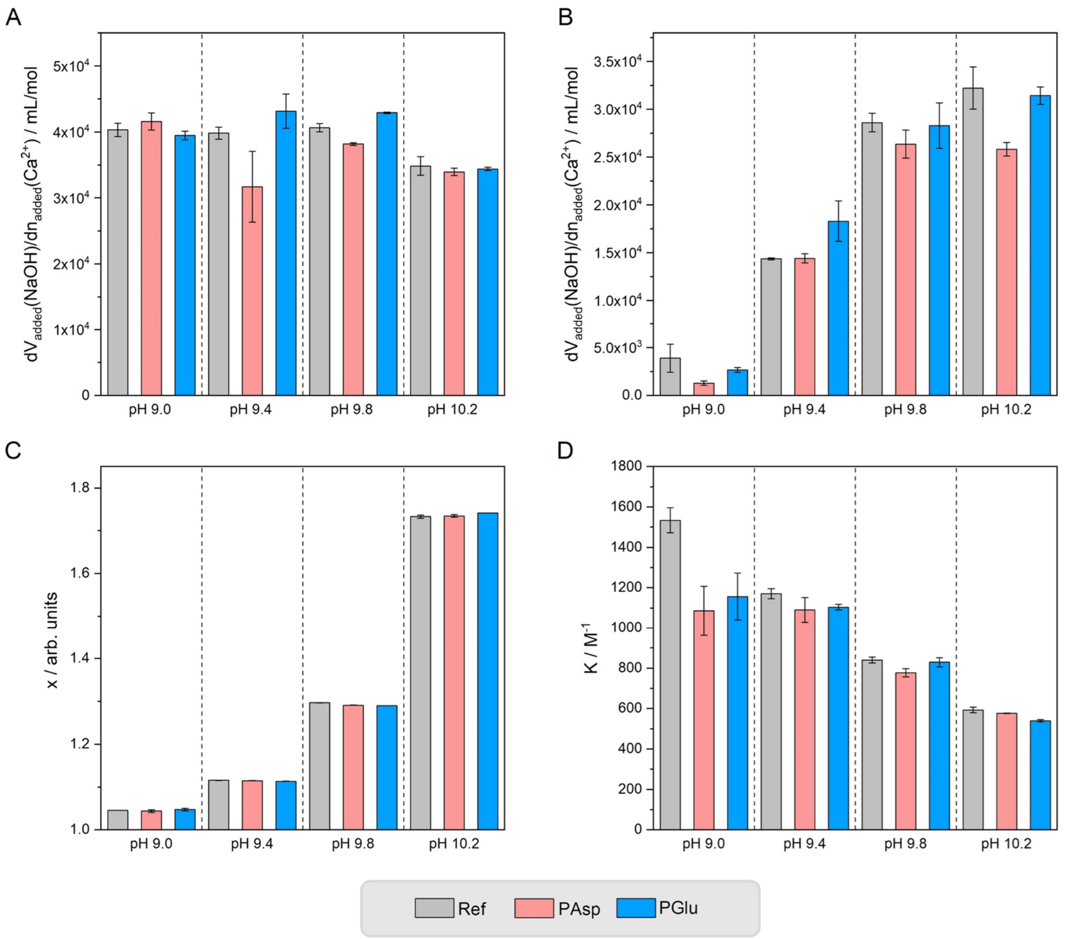

Supplementary Figure 1. Quantitative evaluation of titration experiments. Titration experiments without additive (grey), 10 mg/L PAsp (red) and 10 mg/L PGlu (blue) were evaluated. Numerous parameters were compared in the search for a correlation with the strong polymer- and pH-dependency of nucleation inhibition (Figure 1a in the main text). a) The slope of molar amount of added NaOH per amount of added $CaCl_2$ for the postnucleation regime is shown. For reference experiments, only particle growth is expected at this stage, so similar values are expected across the pH range, i.e., the same amount of carbonate is removed from the buffer equilibrium per amount of Ca^{2+} added. b) Slope of molar amount of added NaOH per amount of added $CaCl_2$ for the prenucleation regime. Although evaluations of the extent of NaOH addition are challenging (see section 2.3.3) and low polymer concentrations of 10 mg/L were used, at three of four pH values (except pH 9.4) a trend in the slopes is visible. Thereby, the reference experiments usually show the highest slope, followed by PGlu and then PAsp, corresponding to the same trend as detected for scale factors (Ref → PGlu → PAsp). A lower slope of NaOH addition seems to correspond to a higher scale factor, which is especially evident at pH 9.0, at which the PAsp shows a high scale factor (Figure 1a in the main text) and a much lower slope in NaOH addition compared to the reference experiment (Figure 1b in the main text). The evaluation of microscopic binding parameters for PNC formation (see section 2.2) showed that the c) x (microscopic number of calcium ions that bind a carbonate ion) as well as d) K (microscopic binding equilibrium constant) did not depend on the polymer type or showed no clear effect correlating the binding inhibition. Additional parameters were quantitatively investigated, e.g., slope of free ion product in the prenucleation regime, but no effect directly corresponded to the trend of nucleation inhibition. Therefore, the NaOH addition in the prenucleation regime was the focus of further studies. Error bars represent ± 1-σ-standard deviation.

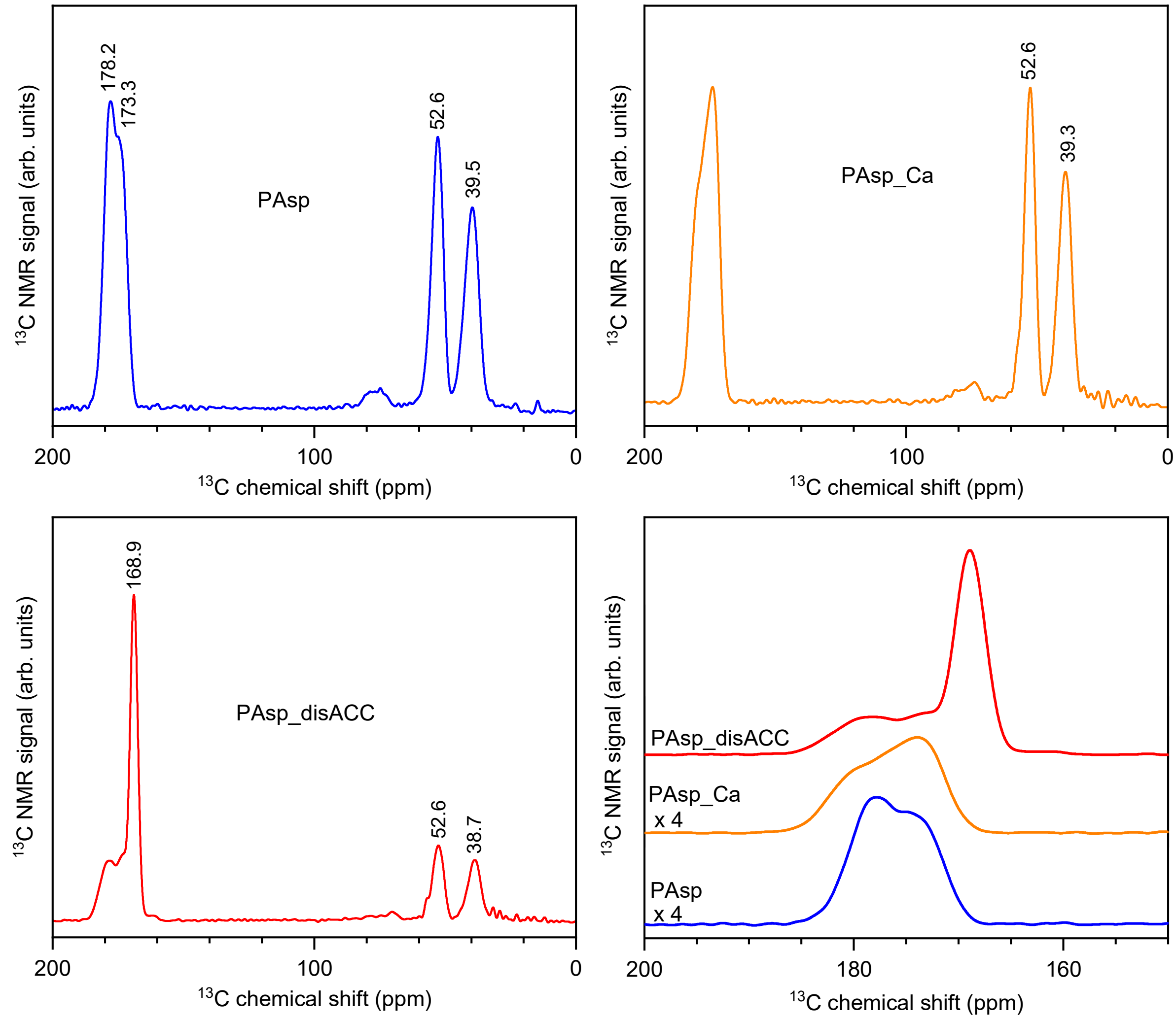

Supplementary Figure 2. Comparison of cross-polarization spectra of PAsp (sodium salt), PAsp calcium salt (PAsp_Ca), and natural abundance PAsp-stabilized ACC (PAsp_disACC) at 10 kHz spinning frequency. The peaks at 52.6 and 39.5 ppm arise from the C_α and C_β of Asp, respectively. Upon ion exchange, the C_α peak is unchanged, but the C_β shifts up field to 39.3 ppm. The C_β shifts further to 38.7 ppm when PAsp is used to stabilize ACC, indicating changes in the conformation of the Asp side chains from interaction with ACC. In the CO-region of PAsp, two resolved peaks are seen, from the CO and C_γ of Asp. Upon ion exchange, the shape of the CO region changes – the two peaks are no longer resolved – and further changes occur in the presence of ACC. In liquid-state NMR, the C_γ is typically observed down-field of the CO.[1,2] A cross peak at 8 and 176 ppm arising from the backbone amines and the carbonyls of PAsp in the FSLG HETCOR spectrum of PAsp-stabilized ACC (Supplementary Figure 3) suggests that this is also the case here. The signal from ACC is centered at 168.9 ppm and has a width of 3.3 ppm, in accordance with previous reports.[3-5]

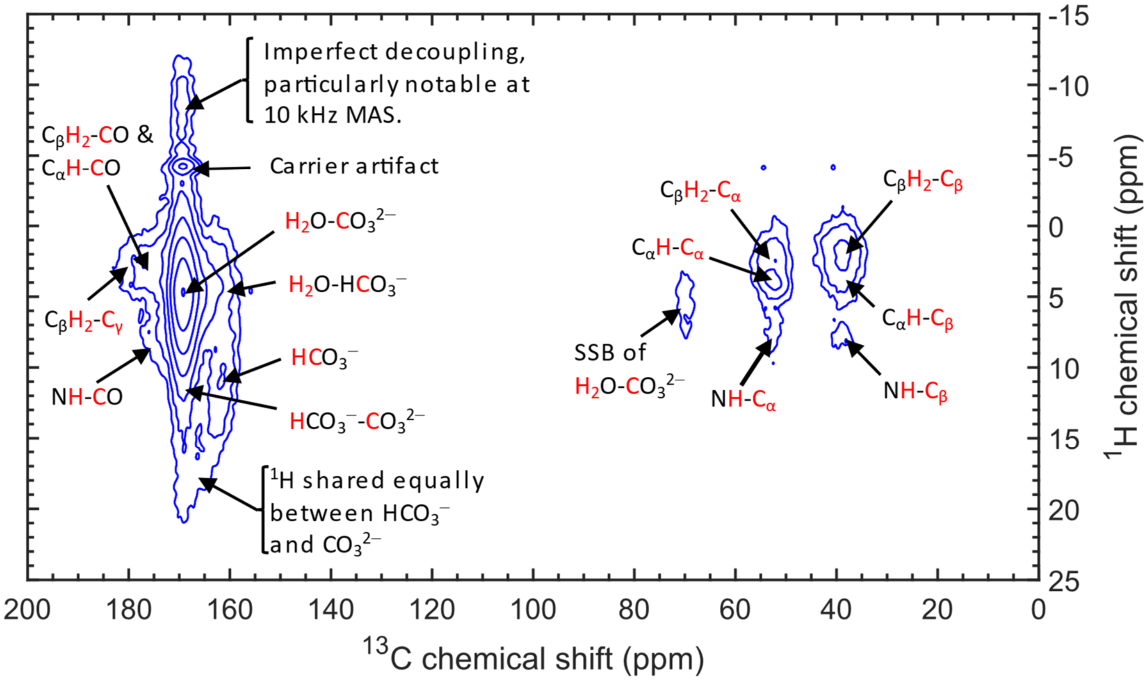

Supplementary Figure 3. FSLG HETCOR spectrum of 100 % ^{13}C-carbonate proto-vaterite (PAsp_ACC, pH 9.8, 100 mM $CaCl_2$ solution added at 0.01 mL/min, see methods section in the main manuscript) ACC stabilized by PAsp. The spectrum was recorded after a contact time of 400 µs, with a spinning frequency of 10 kHz, and at room temperature. Assignments of cross peaks are indicated in the figure.

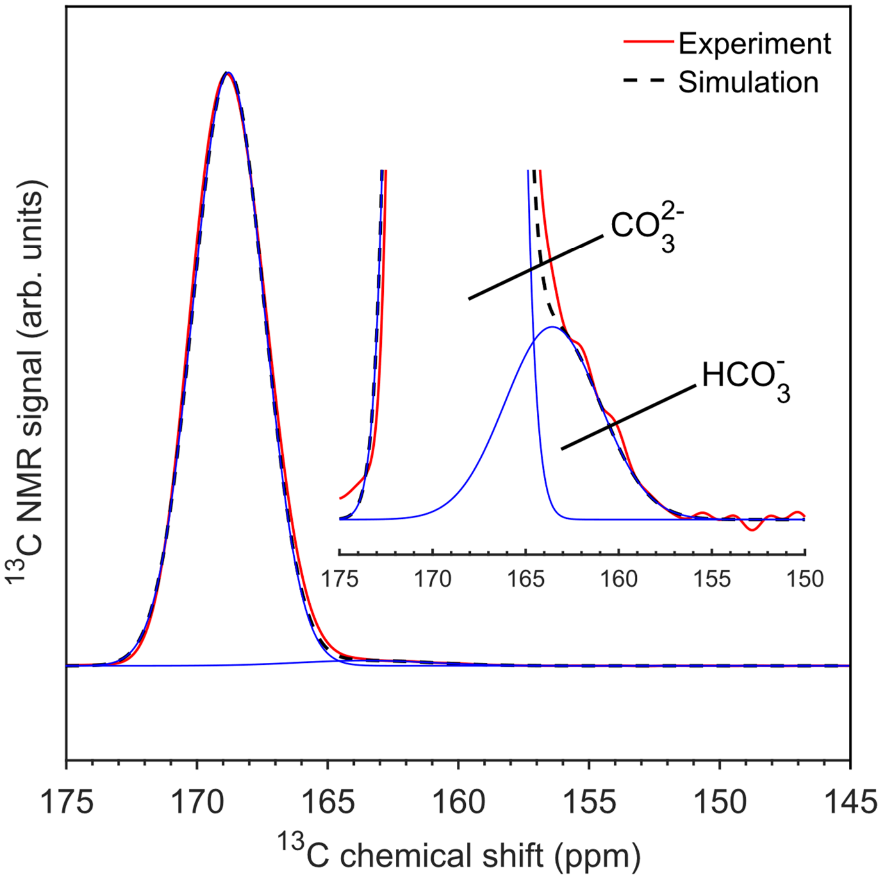

Supplementary Figure 4. Quantitative analysis of the bicarbonate content of PAsp-stabilized ACC (PAsp_disACC, pH 9.8, 200 mM $CaCl_2$ solution added at 0.4 mL/min, see methods section in the main manuscript) from the ^{13}C direct excitation MAS NMR spectrum of a sample prepared with 100 % ^{13}C-carbonate. The spectrum is recorded with a recycle delay of 170 s (corresponding to $1.26 \cdot T_1$ of carbonate), a spinning frequency of 10 kHz, and at room temperature. To determine the width and position of the bicarbonate signal, two Gaussian functions were first fitted to the carbonyl region of the ^{1}H-^{13}C cross polarization spectrum (in which the bicarbonate peak is more pronounced) of the same sample. In the subsequent fit of the ^{13}C direct excitation spectrum, only the amplitudes of the two Gaussian functions were varied. After correction for the longitudinal relaxation times of the two ^{13}C species (about 135 s for carbonate and about 100 s for bicarbonate) a bicarbonate/carbonate ratio of 0.014 is found from the ratio of the intensities of the two Gaussian functions.

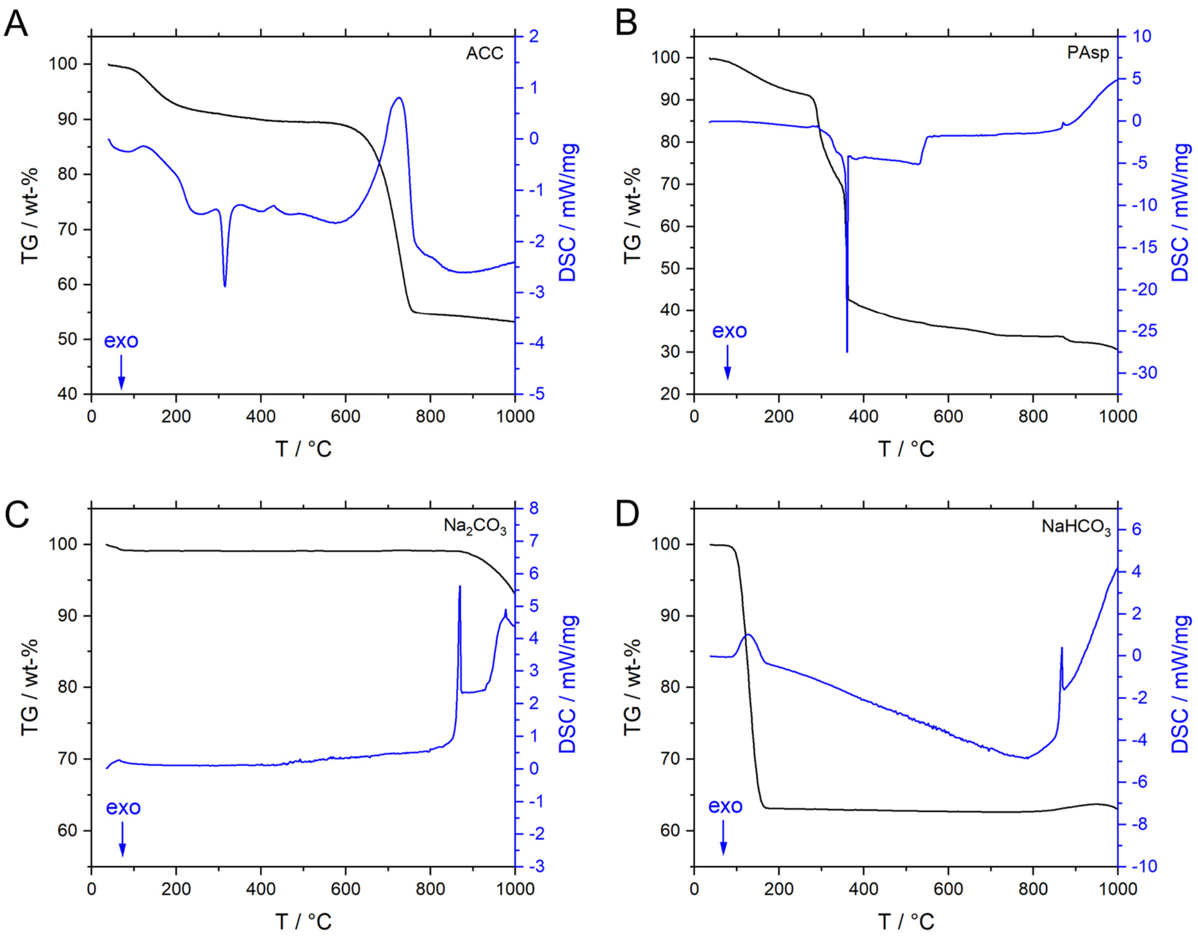

Supplementary Figure 5. TGA and DSC analysis of selected samples. As reference for the polymer-stabilized ACC sample (PAsp_ACC), pure ACC, pure polymer, and sodium (bi)carbonates were investigated using TGA (black lines) and DSC analysis (blue lines). For measurements, oxidative atmosphere (Ar:O_2 80:20 v:v) was used. a) Pure ACC shows initial water loss (<200 °C),[6] followed by exothermic ACC crystallization (~320 °C) and final decarbonization (650-750 °C) to form CaO ($CaCO_3 \rightarrow CaO + CO_2\uparrow$). It needs to be emphasized that although ethanol and acetone were used in the ACC synthesis, these solvents are not present in the dried and investigated samples. No significant amounts of solvents were detected in NMR experiments (see Fig. 2a in the main manuscript) and no vibrational bands of organic solvents were detected in TGA-IR measurements (see Fig. 2e in the main manuscript). Therefore, the weight loss below 200 °C can be attributed to the release of water. b) Pure PAsp shows a similar water loss at the beginning (<200 °C) followed by strong exothermic polymer decomposition (300-360 °C). As the purchased PAsp is a (partial) sodium salt, there is still mass from sodium oxide left at the end of the measurements. c) Na_2CO_3 shows no weight loss in the initial stages of the experiment. At ~900 °C, the melting point is reached, visible by an endothermic signal in DSC. Finally (>900 °C), decomposition takes place ($Na_2CO_3 \rightarrow Na_2O + CO_2\uparrow$). d) $NaHCO_3$ shows an endothermic decomposition at ~180 °C, releasing CO_2 and H_2O (2 $NaHCO_3 \rightarrow Na_2CO_3 + H_2O\uparrow + CO_2\uparrow$).[7] As Na_2CO_3 is formed in this step, the curves then follow the Na_2CO_3 decomposition characteristics (as shown in c).

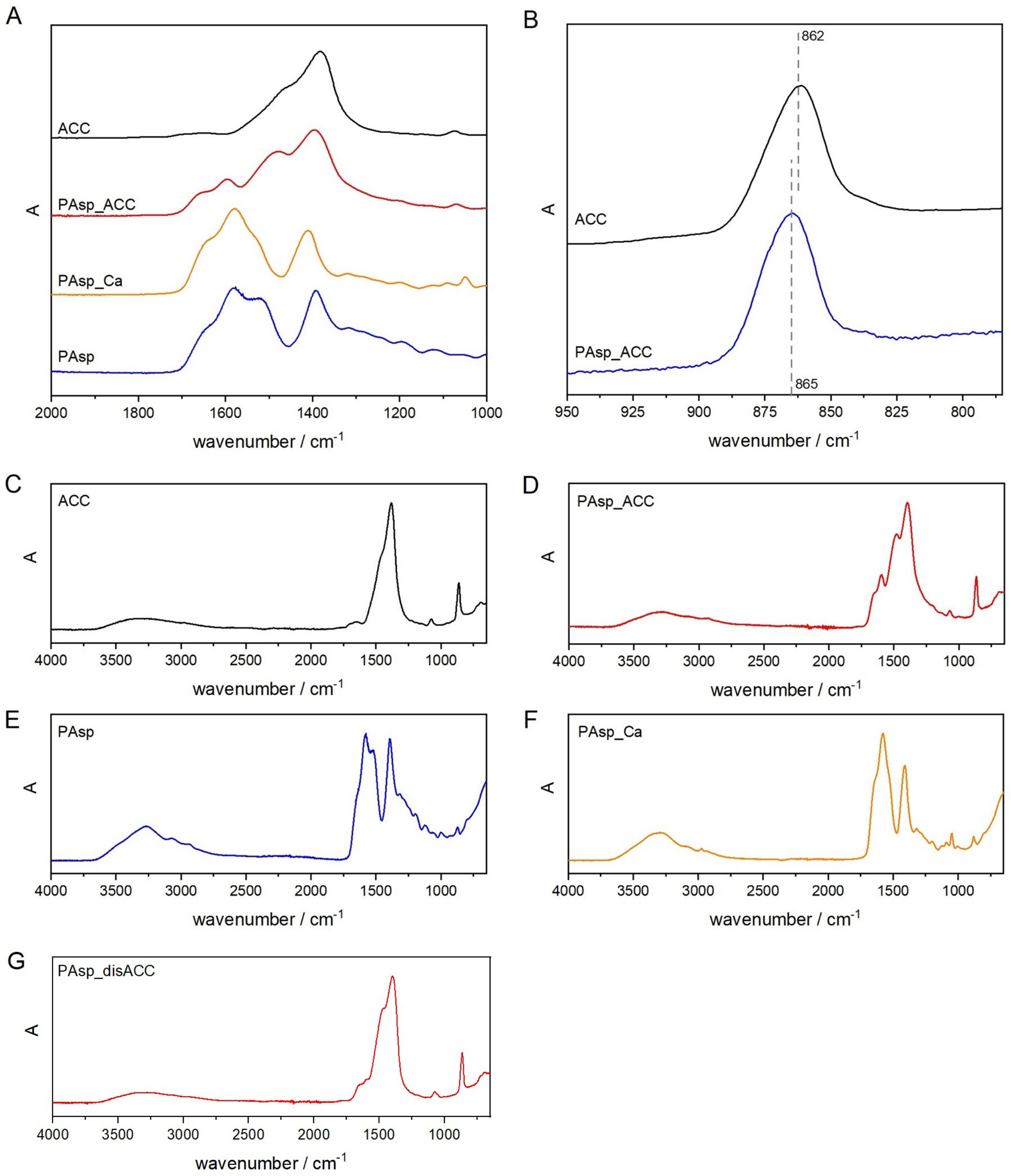

Supplementary Figure 6. ATR-FTIR characterization of polymer and ACC samples. For all samples, the IR spectra of non-^{13}C enriched samples are shown. a) Characteristic vibrations from PAsp/PAsp_Ca are visible in the spectra of the polymer-stabilized ACC sample (PAsp_ACC). b) The calcium carbonate samples are amorphous, as evident from the broad vibrational band at around 863 cm^{-1}. The slight difference between the PAsp_ACC sample (865 cm^{-1}) and the pure ACC samples (862 cm^{-1}) results from different ACC proto-structures.[8] Thereby, the prepared (disordered) ACC exhibits similar spectral features as proto-calcite ACC.[3] c-g) Individual IR spectra for each sample.

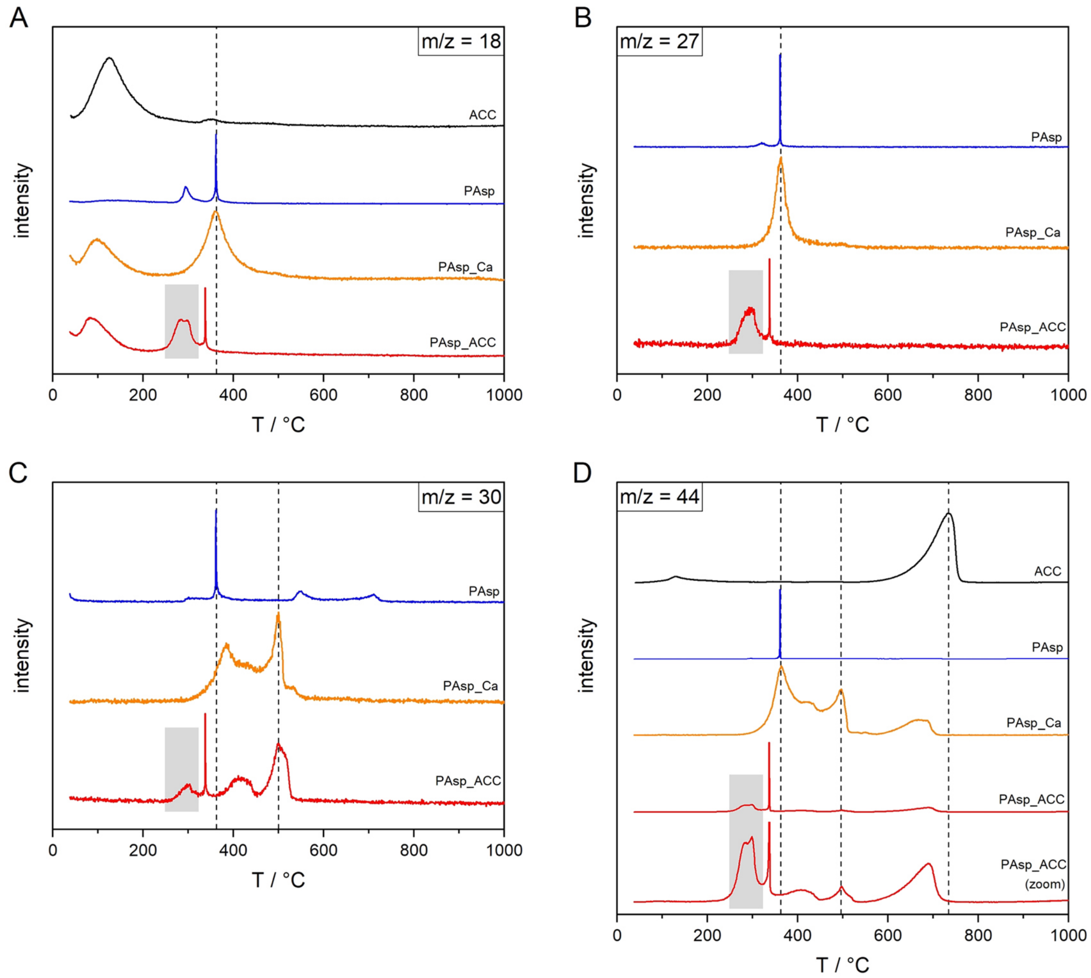

Supplementary Figure 7. TGA-MS analysis of polymer-stabilized ACC and reference samples. The gases released upon decomposition in TGA are analyzed by mass spectrometry. As references for the polymer stabilized ACC (PAsp_ACC, red), the TGA-MS data for pure ACC (black), pure polymer (PAsp, blue) and polymer calcium salt (PAsp_Ca, orange) are shown. The PAsp_Ca sample was synthesized as reference closer resembling the polymer in the mineral phase, i.e., with Ca^{2+} bound to polymer carboxyl groups, as the purchased PAsp is a (partial) sodium salt (synthesis of the samples is described in the methods section in the main manuscript). The curves for m/z with significant amounts of gases detected are shown. a) Results for m/z = 18 (release of H_2O). In addition to the initial release of surface adsorbed and loosely bound water (<200 °C), polymer decomposition (360 °C, dotted line) also results in the release of H_2O. Interestingly, there is a significant broadening for PAsp_Ca compared to the PAsp (sodium salt). The sharp polymer decomposition is also visible in PAsp_ACC, however, in case of PAsp_ACC, a significant release of water is detected prior to the polymer decomposition (250-350 °C, highlighted in grey). As water is released during the decomposition of bicarbonate species (see Supplementary Figure 5d), this fits to the presence of bicarbonate in the stabilized ACC. b) Results for m/z = 27 (release of HCN or $H_2C{=}CH$).[9] The released gases are an indication for polymer decomposition, with nitrogen of HCN arising from the polymer backbone (amide bonds). The sharp polymer decomposition (dotted line) is again visible in all samples, however, in PAsp_ACC, there is significant polymer decomposition detected before the sharp peak (250-350 °C, highlighted in grey). The width and temperature range of this decomposition corresponds well to the detected H_2O release (as shown in a). This indicates that the decomposition of the (polymer-stabilized) bicarbonate species in the ACC structure is triggered by polymer decomposition and/or vice versa. C) Results for m/z = 30 (release of NO, H_2CO, C_2H_6 or $HC{=}NH_2$). In this case, a strong difference between PAsp and PAsp_Ca is detected,

while in PAsp_ACC, features from both samples can be recognized (dotted lines). This shows that in the polymer stabilized ACC sample, aspartic acid segments similar to PAsp_Ca, i.e., with Ca^{2+} bound to the carboxyl group, and segments similar to PAsp, i.e., carboxyl groups not bound to Ca^{2+}, are present. In addition, a decomposition prior to the sharp polymer decomposition is detected (highlighted in grey), confirming the partial destabilization of the polymer by presence of (bi)carbonate species. d) Results for m/z = 44 (release of CO_2). At temperatures of 650-750 °C, $CaCO_3$ decomposition takes place ($CaCO_3 \rightarrow CaO + CO_2\uparrow$), as visible for the pure ACC sample. PAsp shows the familiar sharp decomposition peak, while for PAsp_Ca, several stages of CO_2 release are detected. Especially interesting is the CO_2 release above 600 °C for PAsp_Ca, showing that decomposition of COO-Ca groups proceeds via the formation of $CaCO_3$ as an intermediate.[10] The slight temperature difference for the $CaCO_3$ decomposition between the samples can be explained by the limited high-temperature accuracy of the TGA device. For PAsp_ACC, an overlap of all characteristic signals in reference samples can be detected (dotted lines). In addition, a strong CO_2 release is detected prior to polymer decomposition at 250-350 °C, (highlighted in grey), that we attribute to the decomposition of bicarbonate species as described in the main text. Although the strong release of H_2O and CO_2 in this region fits to the decomposition of bicarbonate species, MAS-NMR analyses prove that only minor amounts of bicarbonate are present in solid ACC. Also, as discussed earlier, a strong interaction of PAsp with calcium (and carbonate species) is detected (as visible in b), so the polymer decomposition characteristics could be changed, resulting in the early release of CO_2 and H_2O. Therefore, additional TGA-IR characterization on ^{13}C enriched samples was performed (Supplementary Figure 9).

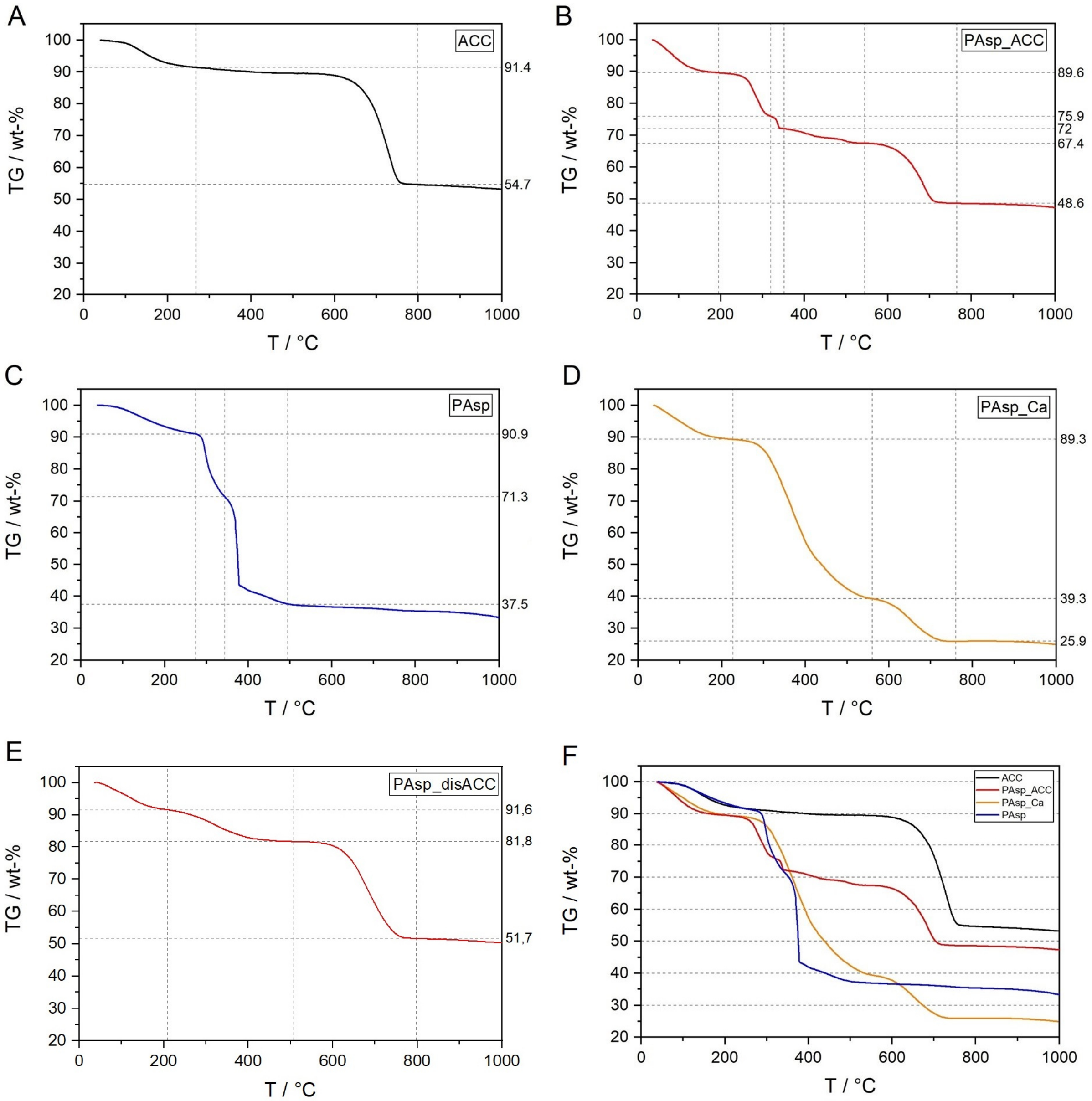

Supplementary Figure 8. TGA characterization of polymer and ACC samples. All measurements were performed in oxidative atmosphere (Ar:O_2 80:20 v/v) and all samples were dried at 40 °C in vacuum prior to measurement. a-e) Individual TGA thermograms for each sample. No significant differences in the thermograms for natural abundance and ^{13}C enriched samples (for MAS NMR investigations) were detected. f) Overlay of all thermograms of samples, showing a significant decomposition via bicarbonate, as discussed in the main text, and polymer present in PAsp_ACC compared to pure ACC, which fits to FTIR analysis (Supplementary Figure 6a).

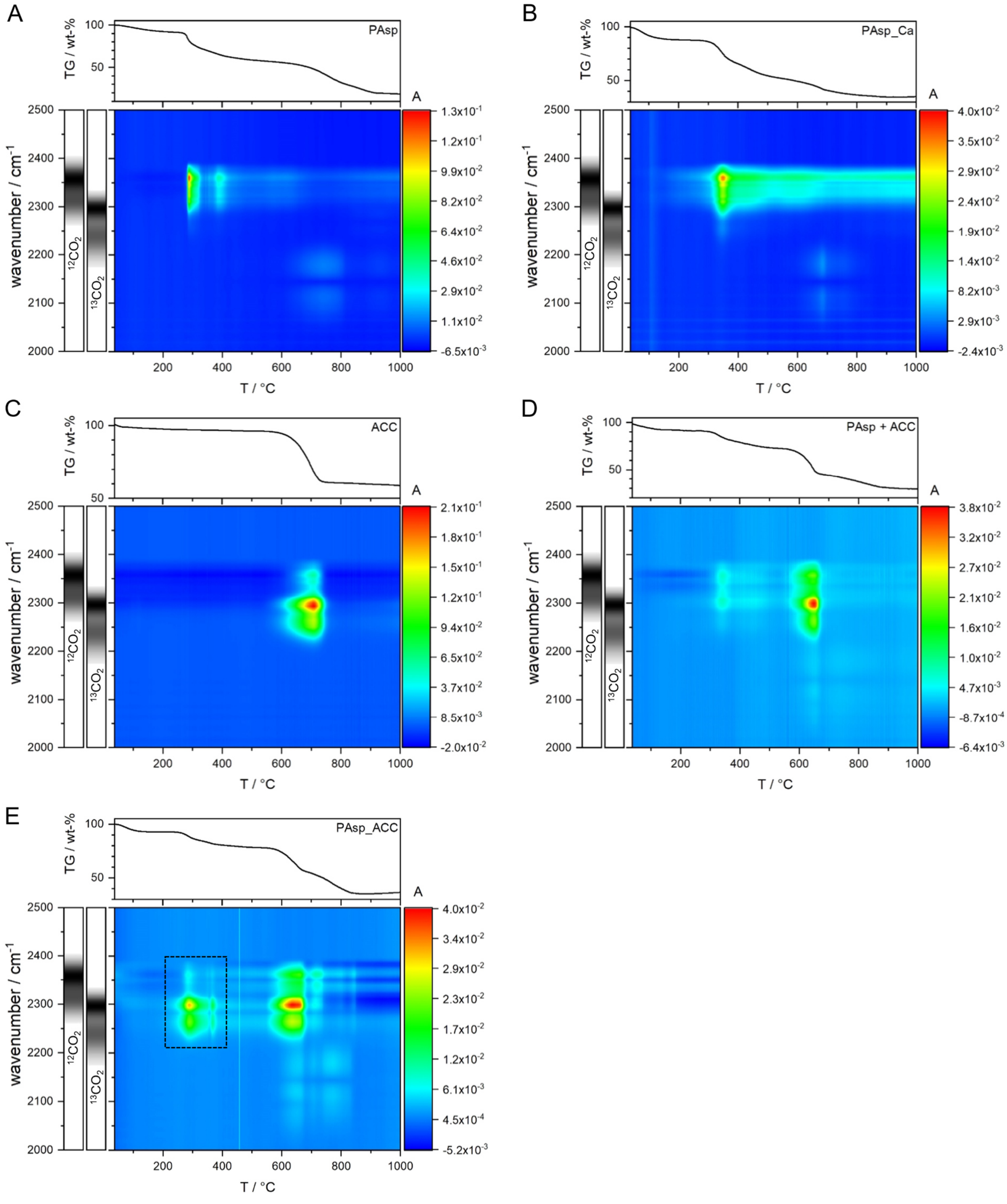

Supplementary Figure 9. TGA-IR analysis of polymer stabilized ACC and reference samples. In addition to mass spectrometry (Supplementary Figure 7), the released gases were analyzed with FTIR spectroscopy to further investigate the structure of the polymer stabilized ACC sample. Especially the characteristics of the strong CO_2 release for PAsp_ACC at around 250-350 °C (Supplementary Figure 7d) is of interest, as we propose that this is caused by decomposition via the formation of bicarbonate species in the sample (see the main text). Therefore, ^{13}C enriched carbonates (99% ^{13}C) were used in the titration experiments, effectively labelling all mineral carbonate species. Simultaneously, regular PAsp, i.e., possessing natural abundance of ^{13}C (99% ^{12}C), was used. In this way, the CO_2 species released during sample decomposition can be distinguished by FTIR spectroscopy, allowing us to determine at which temperatures polymer decomposition (release of mainly $^{12}CO_2$, ν ~ 2275-2400 cm^{-}

^{1}) and decomposition of mineral carbonate species (release of mainly $^{13}CO_2$, ν ~ 2200-2325 cm^{-1}) take place. Herein, the depicted plots show a "top view" on the 3D TGA IR data (3D data of PAsp_ACC is shown Figure 2e in the main text) for the relevant range of wavenumbers for CO_2 detection. a) Pure PAsp shows strong release of $^{12}CO_2$ upon its decomposition at around 300 °C while for b) PAsp_Ca the $^{12}CO_2$ release is broader and also takes place at higher temperature due to the strong interactions of the polymer with Ca^{2+} and formation of $CaCO_3$ as an intermediate (as described in Supplementary Figure 7d). A small release of $^{13}CO_2$ is detected as well, as there is still 1% ^{13}C (natural abundance) present in the polymer. In addition, release of CO species (ν ~ 2000-2200 cm^{-1}) is detected above 600 °C, however, this shall not further be discussed here. c) Pure ^{13}C enriched ACC shows the characteristic $CaCO_3$ decomposition and $^{13}CO_2$ release starting from 600 °C. In this case, a small amount of $^{12}CO_2$ is detected, as the (bi)carbonates used for preparation of the sample are "only" 99% ^{13}C enriched. d) A sample was prepared by mixing the previously discussed samples of pure ^{13}C enriched ACC and pure PAsp (50:50 w/w), which can be used as a reference of a mineral sample with significant amounts of polymer present in the sample. At 300 °C, only $^{12}CO_2$ is released from the polymer decomposition and starting from 600 °C, both $^{12}CO_2$ and $^{13}CO_2$ are released. Thereby, $^{12}CO_2$ arises from the $CaCO_3$ intermediate formed upon decomposition of the polymer,[10] while $^{13}CO_2$ is released from the $CaCO_3$ species formed from ACC crystallization. E) The polymer-stabilized ACC sample synthesized in titration shows a striking difference compared to the simple mixture of ACC and polymer. This time, starting at 250 °C, the main release is $^{13}CO_2$ (dotted box), showing that indeed the decomposition of mineral (bi)carbonate species takes place at this temperature. This also excludes other side reactions that might be responsible for the weight loss in this temperature range, such as formation and decomposition of Ca-polymer coacervate type species, Ca-ethanolate species formed during quenching of the sample in ethanol or weight losses due to (calcium)hydroxide coprecipitation and decomposition, as all these compounds do not involve the release of $^{13}CO_2$ upon decomposition.[11] Also, simple (co)precipitation of $NaHCO_3$ can be excluded due to the exothermic decomposition characteristics of this bicarbonate species (see Figure 2b in the main text, in contrast to the endothermic decomposition of $NaHCO_3$ as shown in Supplementary Figure 5d). In addition, no $NaHCO_3$ was detected in MAS NMR (Figure 2a in the main text), and no sodium oxides were detected in XRD analysis of the precipitate formed after TGA (Supplementary Figure 10).

The difference between PAsp_ACC I and the simple mixture of ACC and PAsp (d) suggests that the ACC decomposes via bicarbonate as discussed in the main text. The weight loss from this bicarbonate species is 13.7% (Supplementary Figure 8b), and assuming decomposition of bicarbonate species according to: 2 HCO_3^- (122 u ↔ 100%) → CO_3^{2-} + H_2O↑ + CO_2↑ (60 u + 18 u↑ + 44 u↑ ↔ 49% + 15%↑ + 36%↑) one can see that twice the amount of bicarbonate is present in the sample compared to the detected weight loss due to release of H_2O and CO_2. According to this calculation, 2·13.7% = 27% bicarbonate forms from calcium deficient carbonates upon release of structural water, as discussed in the main text. As visible in the TGA-MS data, likely some PAsp decomposition takes place in this temperature range as well (Supplementary Figure 7b), potentially triggering or being triggered by bicarbonate decomposition, so the actual amount of bicarbonate generated in the sample upon heating is (slightly) less than 27%. In any case, this amount essentially agrees with the extent of bicarbonate entrapment within the DLP precursor predicted by titration experiments (Figure 1d in the main text).

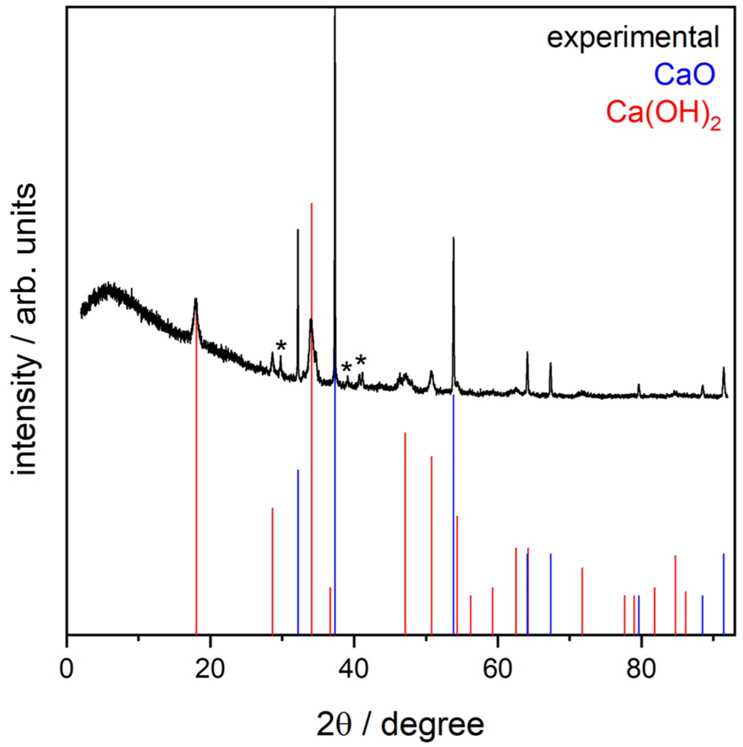

Supplementary Figure 10. Investigation of the sodium content in polymer-stabilized ACC (PAsp_ACC). After TGA analysis (oxidative atmosphere, heating to 1000 °C), the residue was investigated using XRD. No reflections due to sodium oxides are visible, while CaO and $Ca(OH)_2$ are detected as main components.[12,13] According to this data, the presence of sodium salts in the ACC sample, i.e., coprecipitated Na_2CO_3 or $NaHCO_3$, is unlikely. Asterixis correspond to reflections that could not be assigned. The available XRD database (Cambridge Structural Database) was checked to identify these reflections, but no assignment was possible. These reflections were also compared to those of all listed sodium containing compounds (especially oxides that might be formed in TGA) but no compound could be identified.

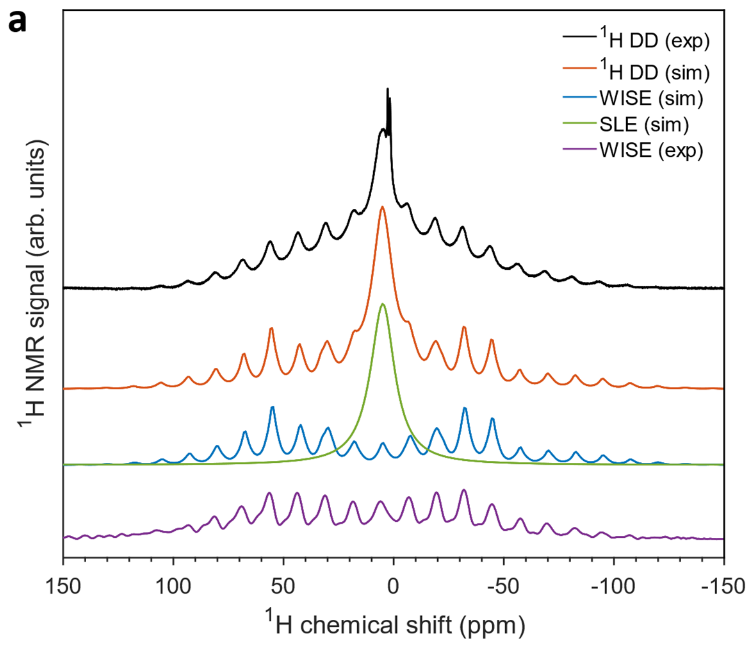

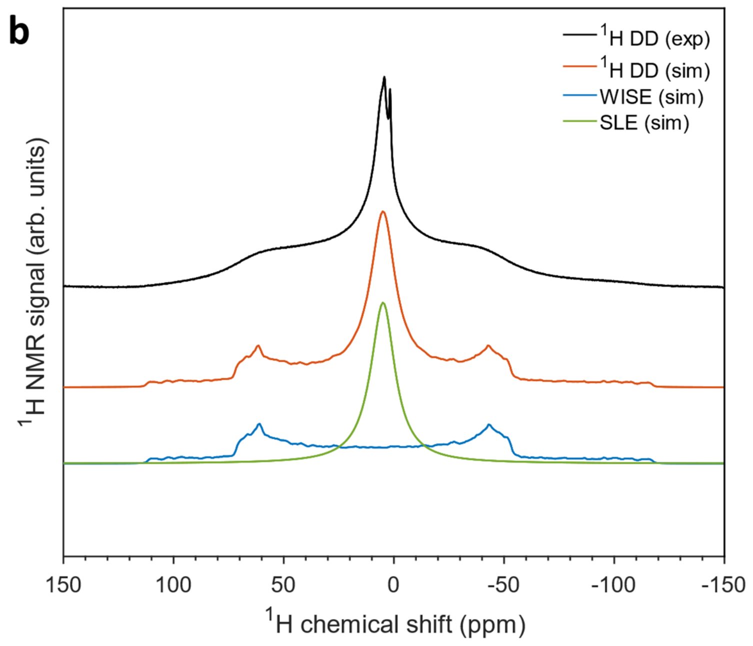

Supplementary Figure 11. Directly and ^{13}C-detected ^{1}H MAS NMR spectra of PAsp-stabilized, 10% ^{13}C-carbonate ACC (PAsp_disACC), with accompanying simulations, at a spinning frequency of 5 kHz (a) and 0 kHz (b) and room temperature. See the text of the main manuscript and the texts accompanying Supplementary Figures 16, 17, and 19 for descriptions of the numerical simulations.

The simulations of the WISE and directly detected spectra (blue and red curves in Figs. 3a and Supplementary Figure 11) somewhat overestimate the strength of the spinning side bands in the regions that coincide with the "horns" of the Pake pattern, both left and right of the central peak. This is likely caused by the amorphous environment of the structural water molecules in ACC. Small variations in the local chemical environment modulate the chemical shift anisotropy and dipolar couplings and lead to a smoothening of the features in the spectra but are not included in the current modelling. This is borne out in the progressive smoothening of the edges of the Pake pattern going from the simulated spectrum at 0 kHz of ACC (Supplementary Figure 11b, blue curve) to the

experimental spectrum of (crystalline) MHC (Supplementary Figure 16, bottom spectrum) to the experimental spectrum of ACC (Supplementary Figure 11b, black curve).

Closer to the central peak, the simulations underestimate the ^{1}H NMR signal. In particular, it seems that the simulation of the isotropic motion does not fully reproduce the broad base of the central peak. While this may partially be attributed to contributions to the spectra from the ^{1}Hs of PAsp (Supplementary Figure 12), we suspect that the main reason is the simplicity of our model, which just concerns one tumbling water molecule. The environment that allows isotropic motion likely consists of multiple water molecules in close proximity. Dipolar couplings between these slow tumbling water molecules are currently not included in the simulations. We note that inclusion of a distribution of correlation times did not improve the match between simulation and experiment, but rather altered the shape of the central peak in an unrealistic manner.

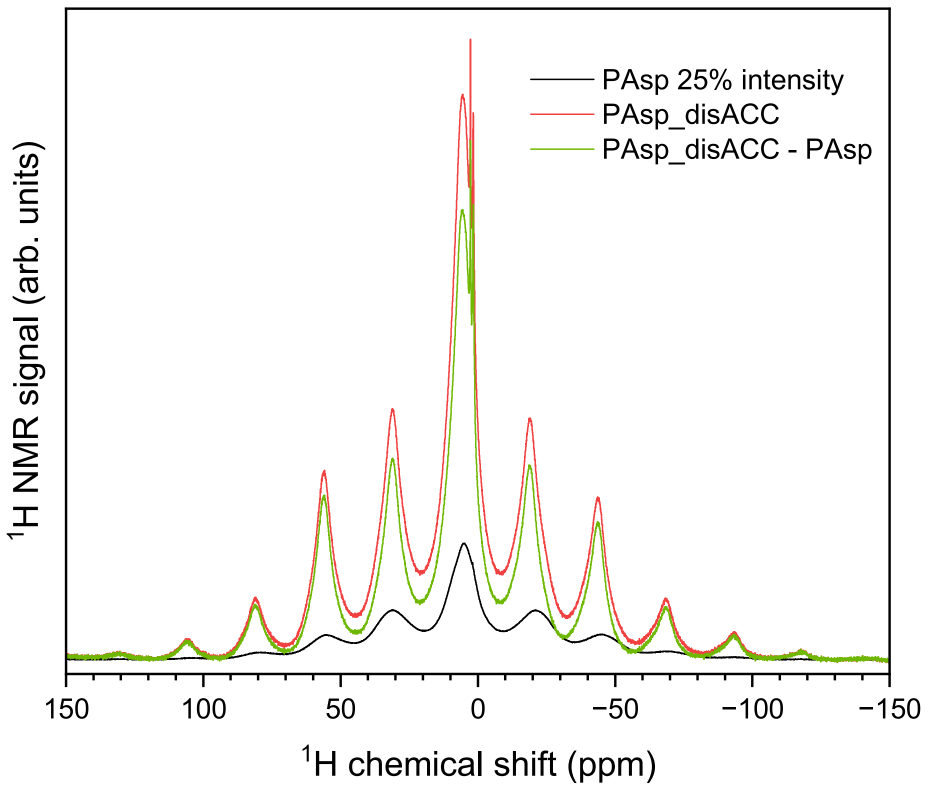

Supplementary Figure 12. Directly detected ^{1}H MAS NMR spectra of 10% ^{13}C-CO_3^{2-} PAsp-stabilized ACC (PAsp_disACC) and of pure PAsp, both at room temperature and at a spinning frequency of 10 kHz. The intensity of the PAsp spectrum is scaled to 25 % of the intensity of the PAsp-stabilized ACC and subtracted.

In our hands, due to the centrifugal forces, samples of pure ACC show signs of crystallization within hours of spinning. Samples of ACC stabilized by PAsp, however, remained amorphous for many months. The ^{1}Hs of PAsp do contribute to the directly detected ^{1}H spectra. To assess this contribution, first, the PAsp content of a sample was determined by comparing the intensities of the carbonate and C_{α} signals in the ^{13}C direct-excitation spectrum. After correction for the long T_{1n} of carbonate, a polymer content of 16% by weight was found for the shown sample. Assuming 2 ^{1}Hs per carbonate and 4 ^{1}Hs per Asp unit, the ^{1}Hs of PAsp contribute 25% of the total ^{1}H NMR intensity. The intensity of a ^{1}H NMR spectrum of PAsp, which was separately measured under the same conditions, was scaled to make up 25% of the intensity of the PAsp-stabilized ACC spectrum and subtracted. The result is shown in the figure and indicates that the ^{1}Hs of PAsp do not significantly alter the shape of the spectrum.

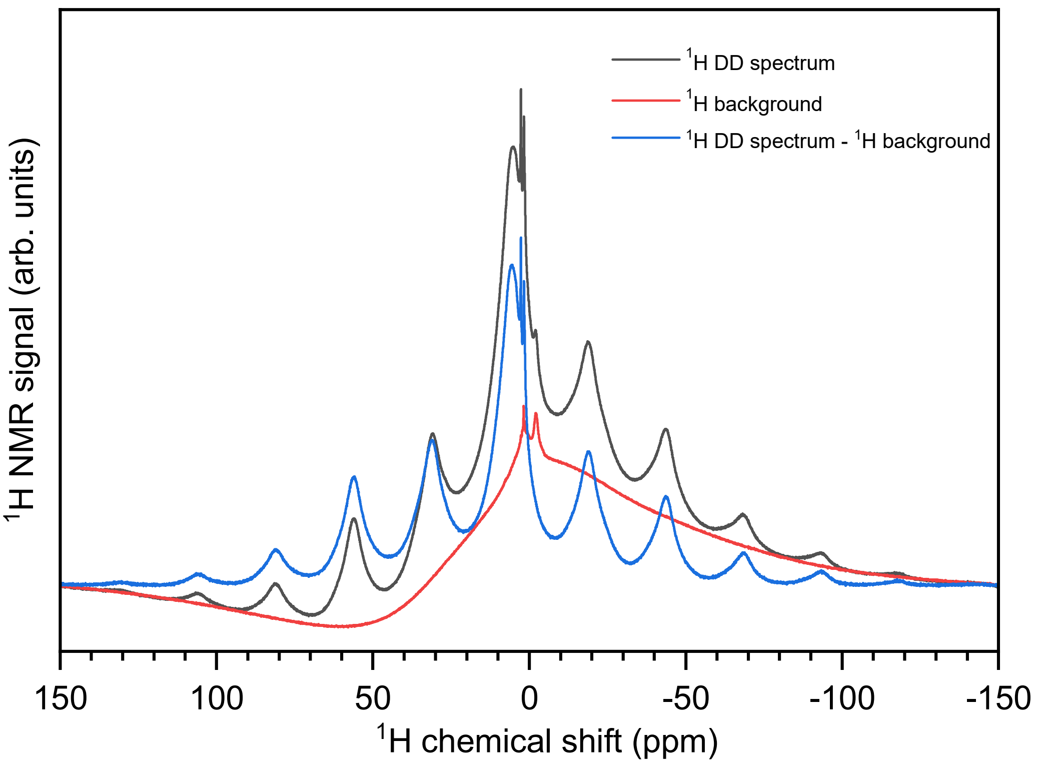

Supplementary Figure 13. The MAS NMR probe gives rise to a broad background signal, which overlaps with the ^{1}H signals from the samples. This background signal was measured separately using a rotor filled with KBr and the same temperature settings as used for the sample spectra. The same phase correction was applied to the background spectrum as to the sample spectra, after which the background spectrum was subtracted from the sample spectra, as illustrated. After subtraction of the ^{1}H background, the ^{1}H spectrum arising solely from the sample is obtained, in this case for 10 % ^{13}C-carbonate PAsp-stabilized ACC (PAsp_disACC). The spinning frequency was 10 kHz.

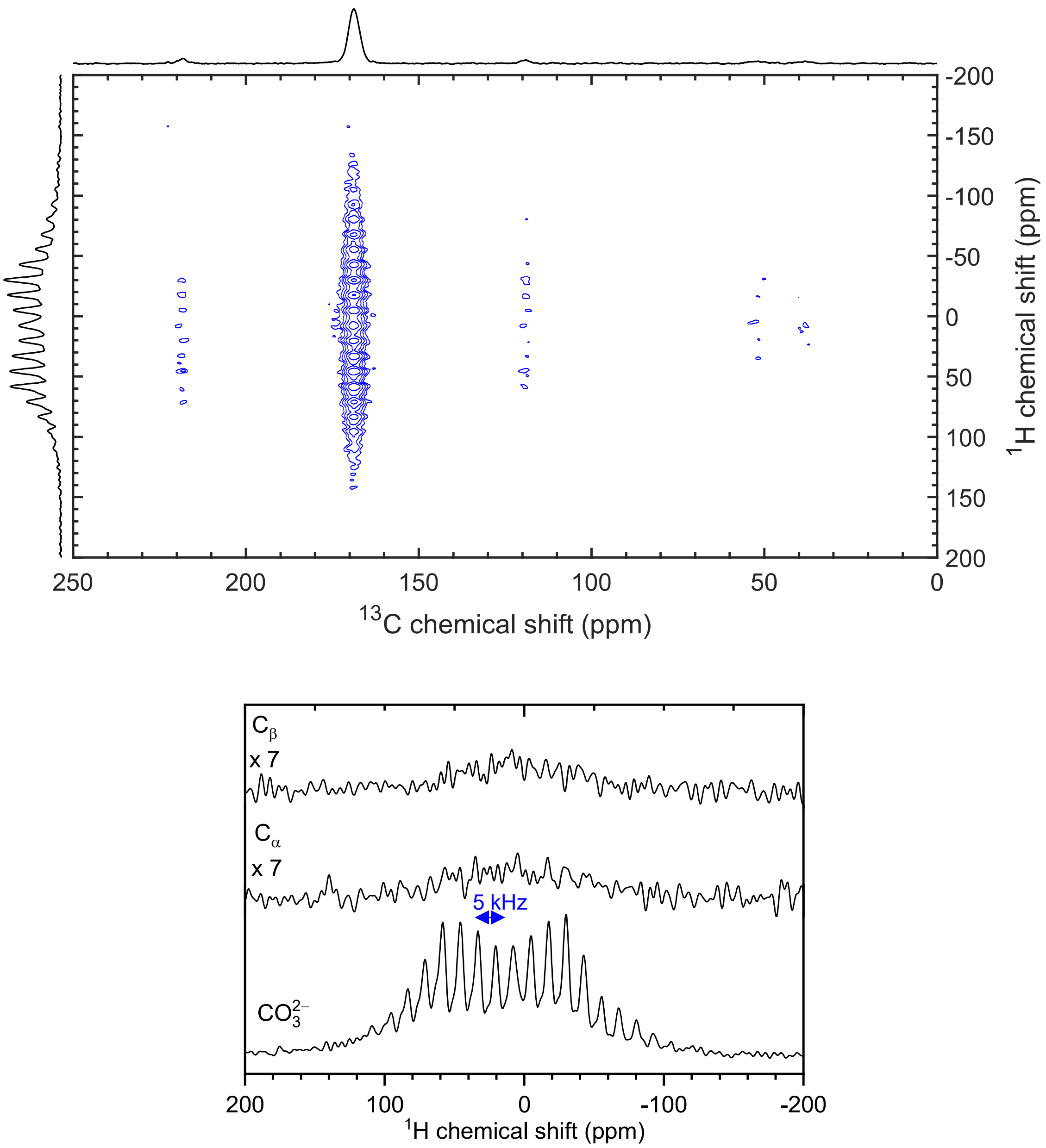

Supplementary Figure 14. (top) WISE spectrum of 10 % ^{13}C-carbonate PAsp-stabilized ACC (PAsp_disACC) at 5 kHz spinning frequency. Projections along the ^{13}C and ^{1}H dimensions are shown on the left and at the top, respectively. The lowest contour level is set at 3 times the root-mean-square of the noise. The height of the contour levels is incremented by a factor of 1.7. (bottom) Summations over vertical slices covering the cross-peaks of the C_β, C_α of Asp, and carbonate species of ACC. In the ACC-carbonate slice, the Pake pattern of structural water (horn separation 100-110 ppm) is visible through the spinning-sidebands. No sidebands are discernable in the C_β and C_α slices.

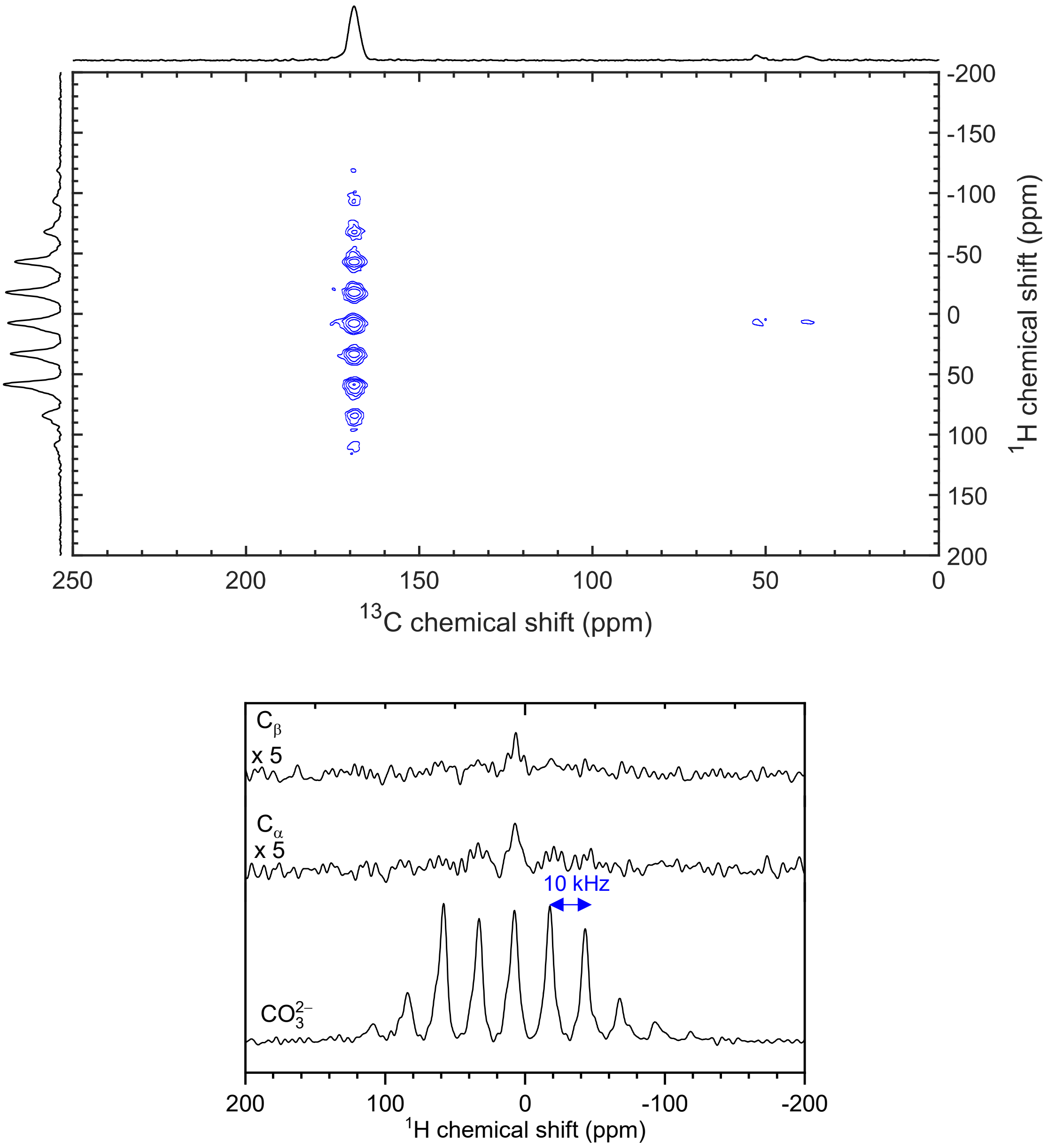

Supplementary Figure 15. (top) WISE spectrum of 10 % ^{13}C-carbonate PAsp-stabilized ACC (PAsp_disACC) at 10 kHz spinning frequency. Projections along the ^{13}C and ^{1}H dimensions are shown on the left and at the top, respectively. The lowest contour level is set at 3 times the root-mean-square of the noise. The height of the contour levels is incremented by a factor of 1.625. (bottom) Summations over vertical slices covering the cross-peaks of the C_β, C_α of Asp, and carbonate species of ACC. In the ACC-carbonate slice, the Pake pattern of structural water (horn separation 100-110 ppm) is visible through the spinning-sidebands.

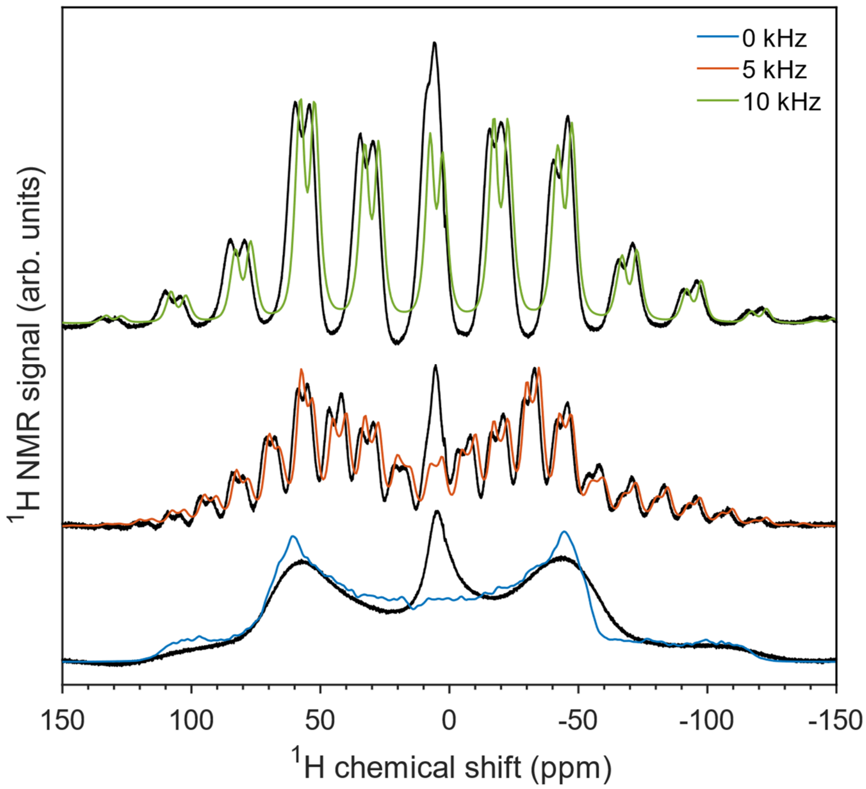

Supplementary Figure 16. Directly detected ^{1}H NMR spectra of monohydrocalcite at spinning frequencies of 0, 5, and 10 kHz and room temperature (black: experiments; blue, red, green: simulations). The ^{1}H NMR background signal from the probe was removed following the procedure outlined in Supplementary Figure 13. Monohydrocalcite was synthesized as described in the methods section in the main manuscript. Numerical simulations make use of the kernel of the magnetic resonance simulation package Spinach.[14]

To simulate the directly detected ^{1}H MAS NMR spectra of monohydrocalcite, the results of electronic structure calculations performed by Huang et al. were used.[15] These calculations were performed using the plane-wave pseudopotential approach within Kohn-Sham density functional theory as implemented in the program CASTEP.[16] The PBE functional was used and the cutoff energy was set to 800 eV. The optimized structure of the unit cell of monohydrocalcite and the calculated chemical shift tensors of the 18 ^{1}H nuclei were imported into Spinach. The isotropic chemical shifts were adjusted using the mineral nahcolite as a reference.[15] Evolution of the ^{1}H magnetization in the x,y-plane was calculated using the Fokker-Planck MAS formalism and a spherical grid.[17] Product states between up to 3 spins were considered in the calculation. Before Fourier transformation, an exponential window function was applied. The simulation script has been added to the example set of the Spinach library at https://spindynamics.org.

The numerical simulations reproduce the experimental spectra remarkably well, including the asymmetry of the Pake pattern and the splitting of the spinning side bands. Compared to the simulation, the experimental spectrum at 0 kHz is more "smooth". This is due to small variations in the structure across the monohydrocalcite microcrystals, which are not included in the numerical simulations. An additional peak at the center of the Pake pattern arises from adsorbed water on the surfaces of the microcrystals. Its intensity decreases with prolonged spinning of the rotor (by a dry N_2 gas stream), but is recovered by exposure of the rotor to the atmosphere of the laboratory.

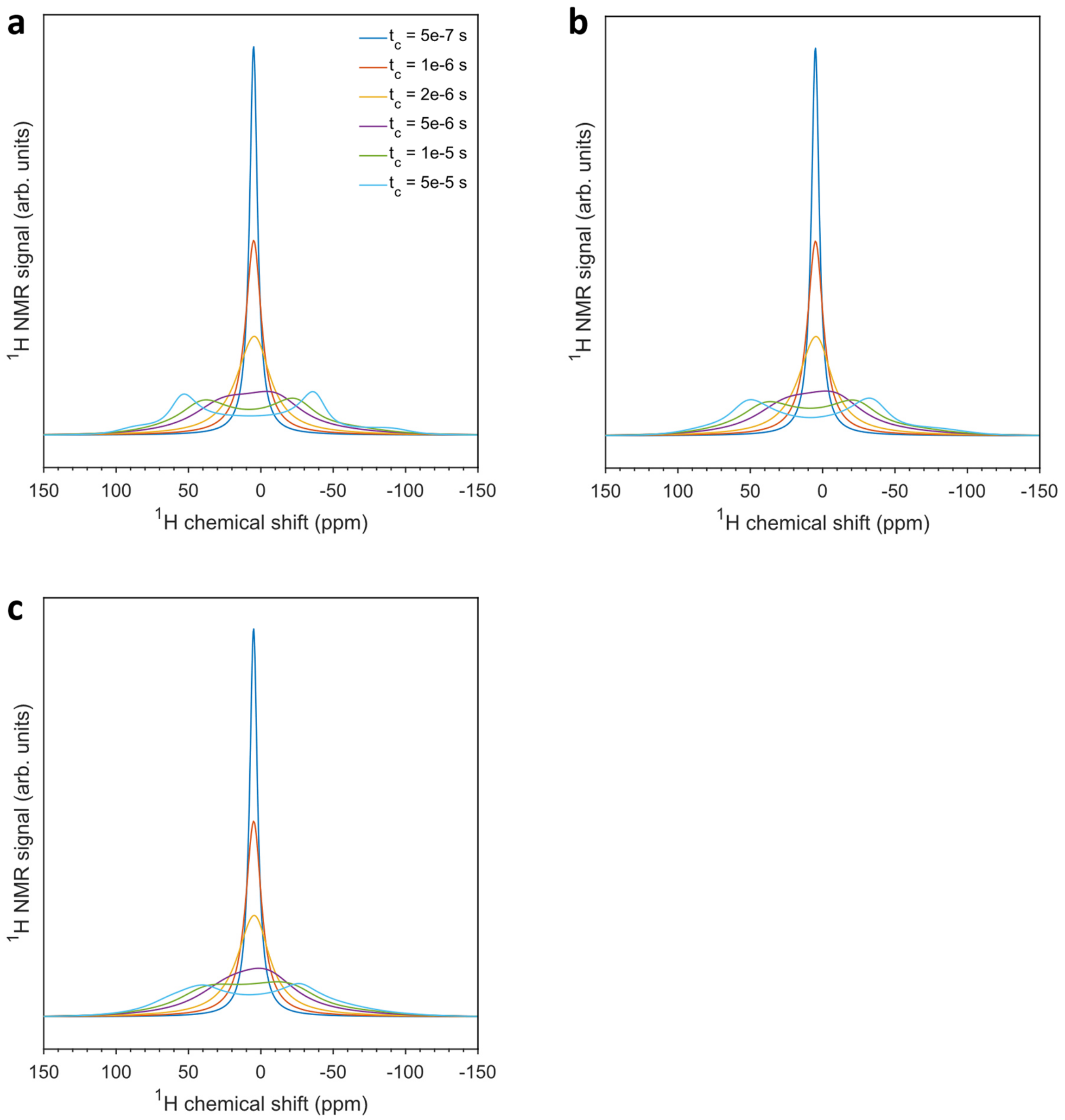

Supplementary Figure 17. Numerically simulated ^{1}H NMR spectra of a water molecule undergoing slow isotropic motion at MAS spinning frequencies of (a) 0 kHz, (b) 5 kHz, and (c) 10 kHz. For the simulations, the magnetic properties of ^{1}Hs #1 and #4 of the monohydrocalcite unit cell were considered. The effects of slow isotropic motion were modelled using the stochastic Liouville equation.[17,18] When rotation correlation times become shorter than $5 \cdot 10^{-6}$ s, the line shape is no longer affected by the magic angle spinning frequency. The simulation script has been added to the example set of the Spinach library at https://spindynamics.org.

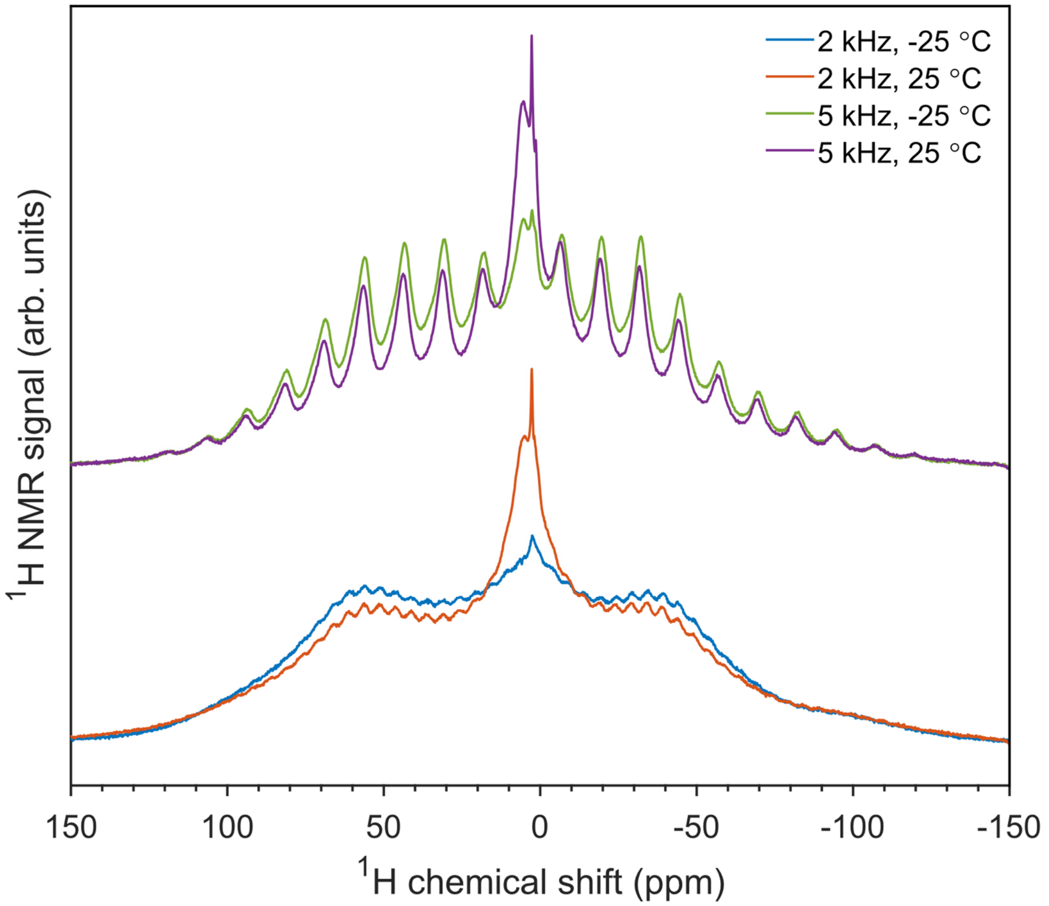

Supplementary Figure 18. Directly detected ^{1}H MAS NMR spectra of 10% $^{13}C\text{-}CO_3^{2-}$ PAsp-stabilized ACC (PAsp_disACC) at 2 and 5 kHz and temperatures of 25 and -25 °C.

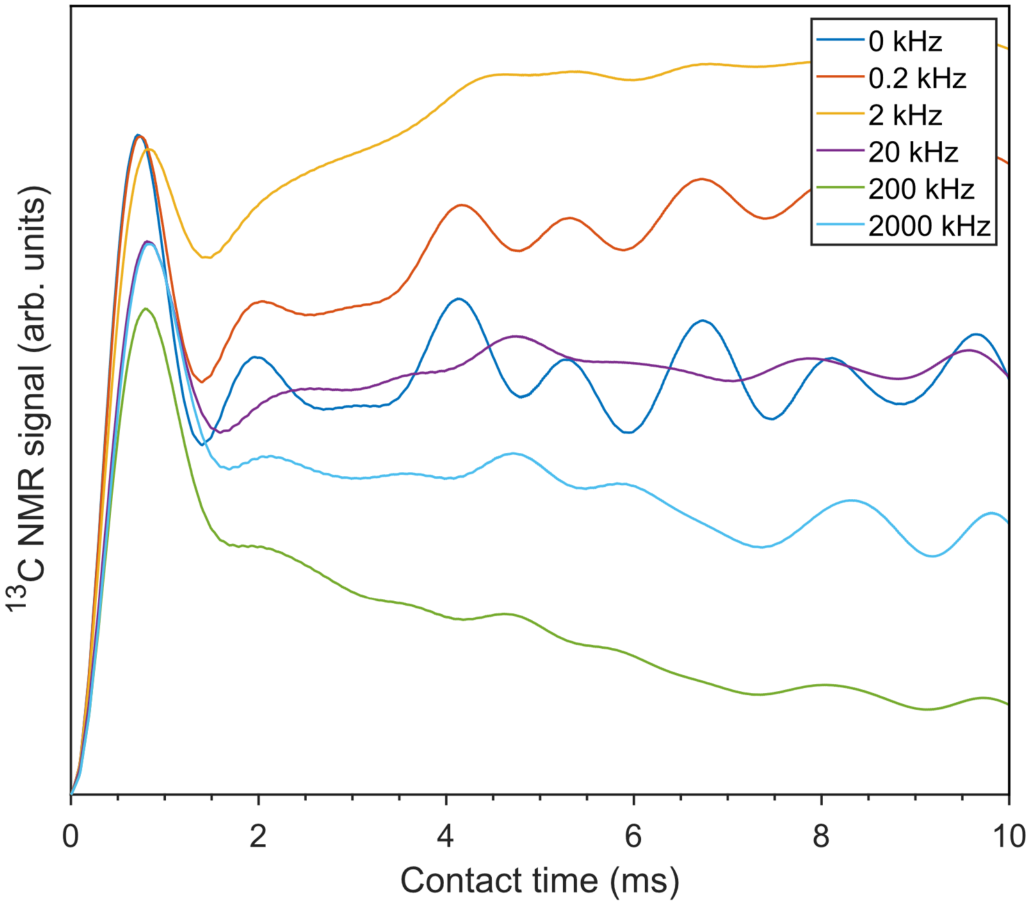

Supplementary Figure 19. Numerical simulations show the effect of a water molecule undergoing 180° flips, at various rates, on the magnetization transfer in a ^{1}H-^{13}C cross polarization experiment. The transfer becomes particularly inefficient at a flipping rate of 200 kHz.

For the simulations shown in the figure, the magnetic properties of ^{1}Hs #1 and #4 and of ^{13}C #19 of the monohydrocalcite unit cell were considered. To model the 180° flips of the water molecule, the Spinach chemical kinetics module was used. The figure shows that, depending on the rate at which the flipping takes place, the magnetization transfer during the cross-polarization contact period can become rather inefficient. The simulations script has been added to the example set of the Spinach library at https://spindynamics.org.

A spin system consisting of ^{1}H #1 and #4 of the monohydrocalcite unit cell and the chemical kinetics module of Spinach were also used to simulate the indirectly detected ^{1}H spectra of ACC (Figure 3a, Supplementary Figure 11, blue curves). The modulation of the chemical shift anisotropy caused by the interchanging of the ^{1}Hs of the water molecule removes the splitting in the spinning sidebands – at a flipping rate of about 5 kHz the shape of the simulated spinning side bands is in agreement with the experimental shape.

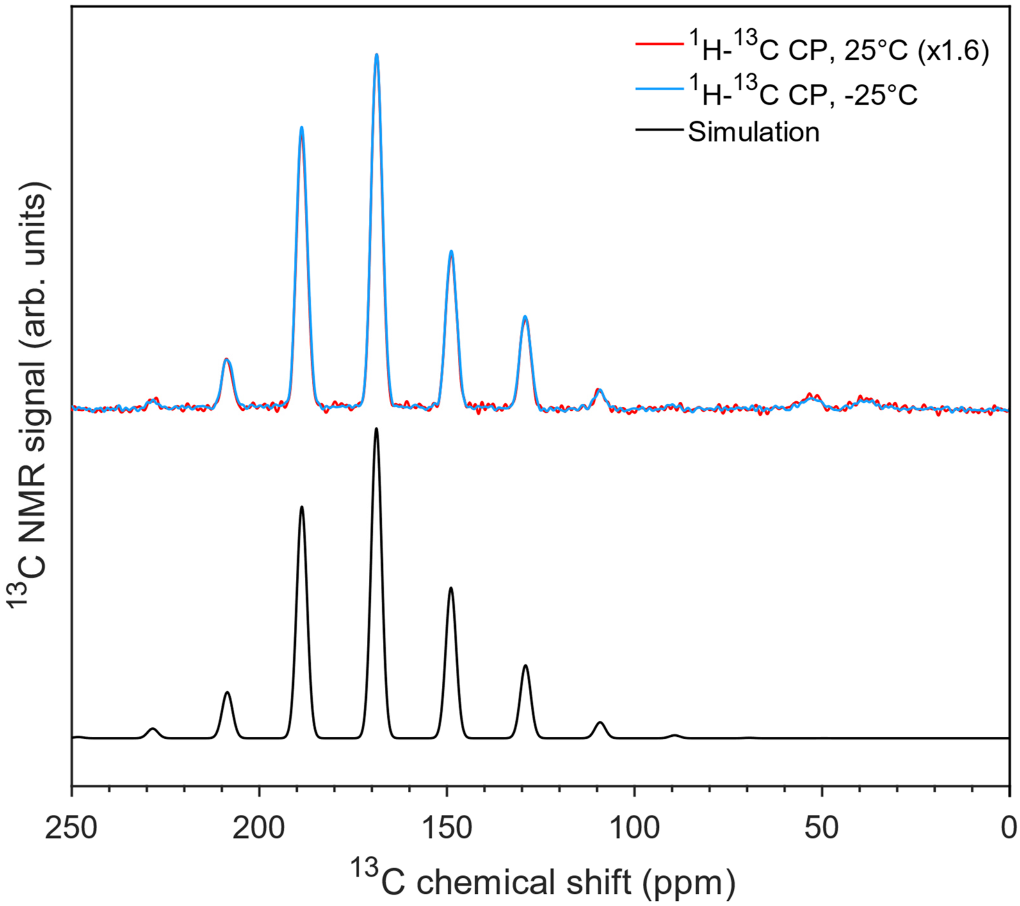

Supplementary Figure 20. ^{1}H-^{13}C cross-polarization spectra of PAsp-stabilized ACC at 2 kHz spinning frequency. Experimental ^{1}H-^{13}C cross-polarization spectra of 10% ^{13}C-CO_3^{2-} PAsp-stabilized ACC (PAsp_disACC) at a spinning frequency of 2 kHz and temperatures of 25 (red) and -25 °C (blue). Spectra were simulated using the following parameters for the chemical shift anisotropy of the ^{13}C nucleus of carbonate in ACC: δ_{iso} = 168.9 ppm, Δ = -49.3 ppm, η = 0.2-0.5 (black). To reproduce the experimentally observed line shape, a Gaussian window function ($e^{-10^{4}\cdot x^{2}}$) was applied to a free induction decay of 8096 points and a dwell time of 20 µs. Anisotropy parameters are in agreement with those reported for carbonate in ACC by Sen et al.[19] (following the Haeberlen convention, the sign of Δ is negative) and for carbonate in a frozen solution of carbonate with pH 7 by Ramnarain et al.[20]

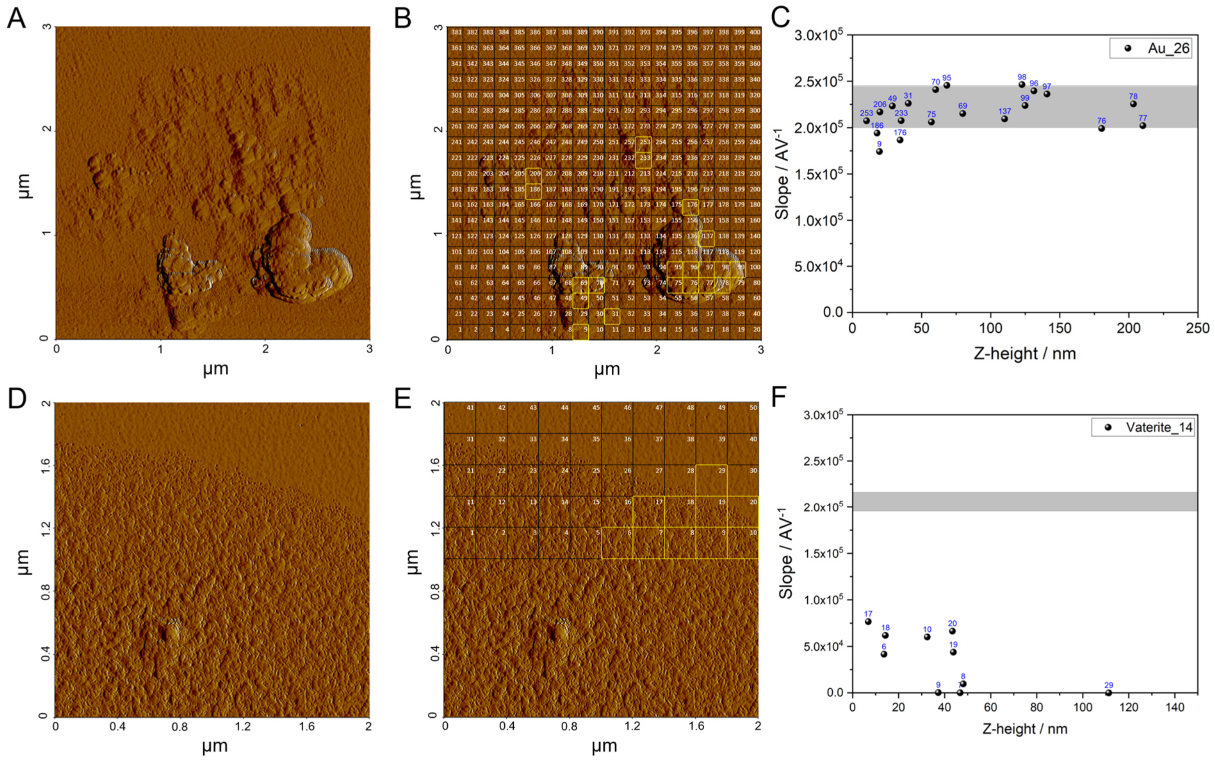

Supplementary Figure 21. C-AFM investigation of reference samples. Determination of particle conductivity was carried out as described in section 4. For method validation, conductive and non-conductive nanoparticles were investigated (synthesis see methods section in the main manuscript). a) Non-contact mode (NCM) amplitude map of a measurement of Au nanoparticles and b) amplitude map overlayed with the 20 x 20 grid of points measured in spectroscopy mode. In yellow, the data points considered for evaluation are highlighted. c) Plot of measured slope of the I/V diagram for each selected data point in dependence of the starting Z-height of the spectroscopy measurement. The area highlighted in gray corresponds to the average value determined for measurements on the Au substrate (standard deviation of at least 30 evaluated points), indicating the maximum conductivity that can be determined. It is evident that Au particles show good conductivity (similar to the Au substrate) over the whole range of measured Z-heights. d-f) Corresponding measurements on vaterite nanoparticles as reference for non-conductive $CaCO_3$ nanoparticles. The results show that the particles possess a lower conductivity compared to the wafer, however still a non-zero slope is detected for particles smaller than 40 nm. This might be due to leakage current across the extremely small Z-heights. Therefore, attention needs to be paid for evaluating particles with Z-height below 40 nm.

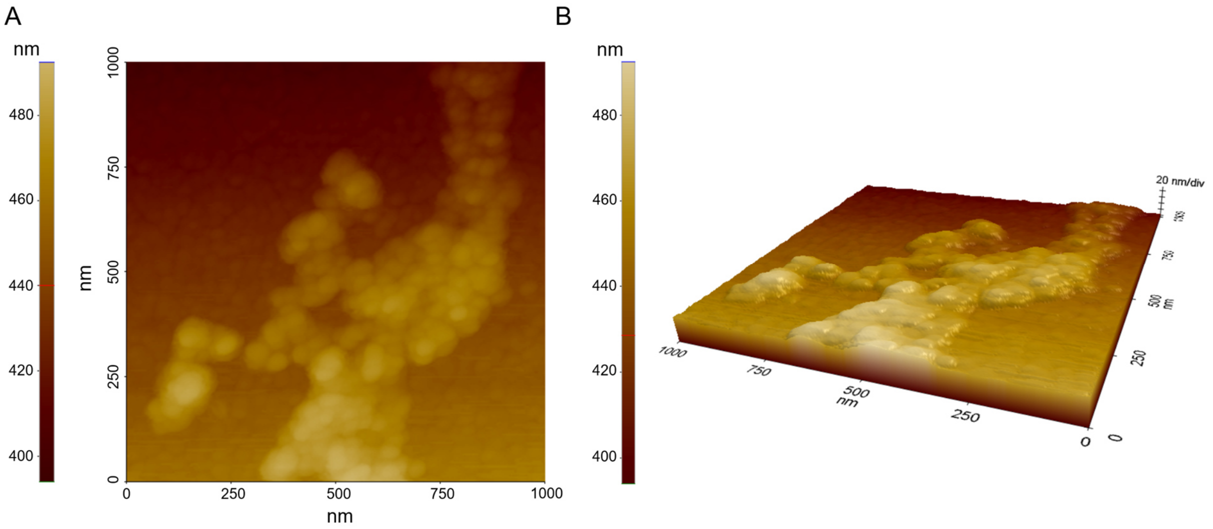

Supplementary Figure 22. AFM height map of polymer-stabilized ACC (PAsp_ACC) particles. The image was recorded in non-contact mode (NCM) and shows the same area used for C-AFM investigations (discussed in Figure 4a,b in the main manuscript and Supplementary Figure 4a-c). a) AFM height map, showing individual ACC particles and agglomeration of particles. b) 3D visualization of the area, using isotropic axis scaling. Due to the gel-like properties of (polymer-stabilized) ACC, the particles spread on the substrate upon precipitation and drying, resulting in an oblate structure. Therefore, although the individual particles show a size of 20-50 nm in top view (a), they possess a height of significantly less than 20 nm. In addition, the samples were prepared using spin-coating, which exerts additional forces on the particles. Similar effects of shape-anisotropy of sedimented amorphous calcium carbonate particles were described in earlier studies.[21,22]

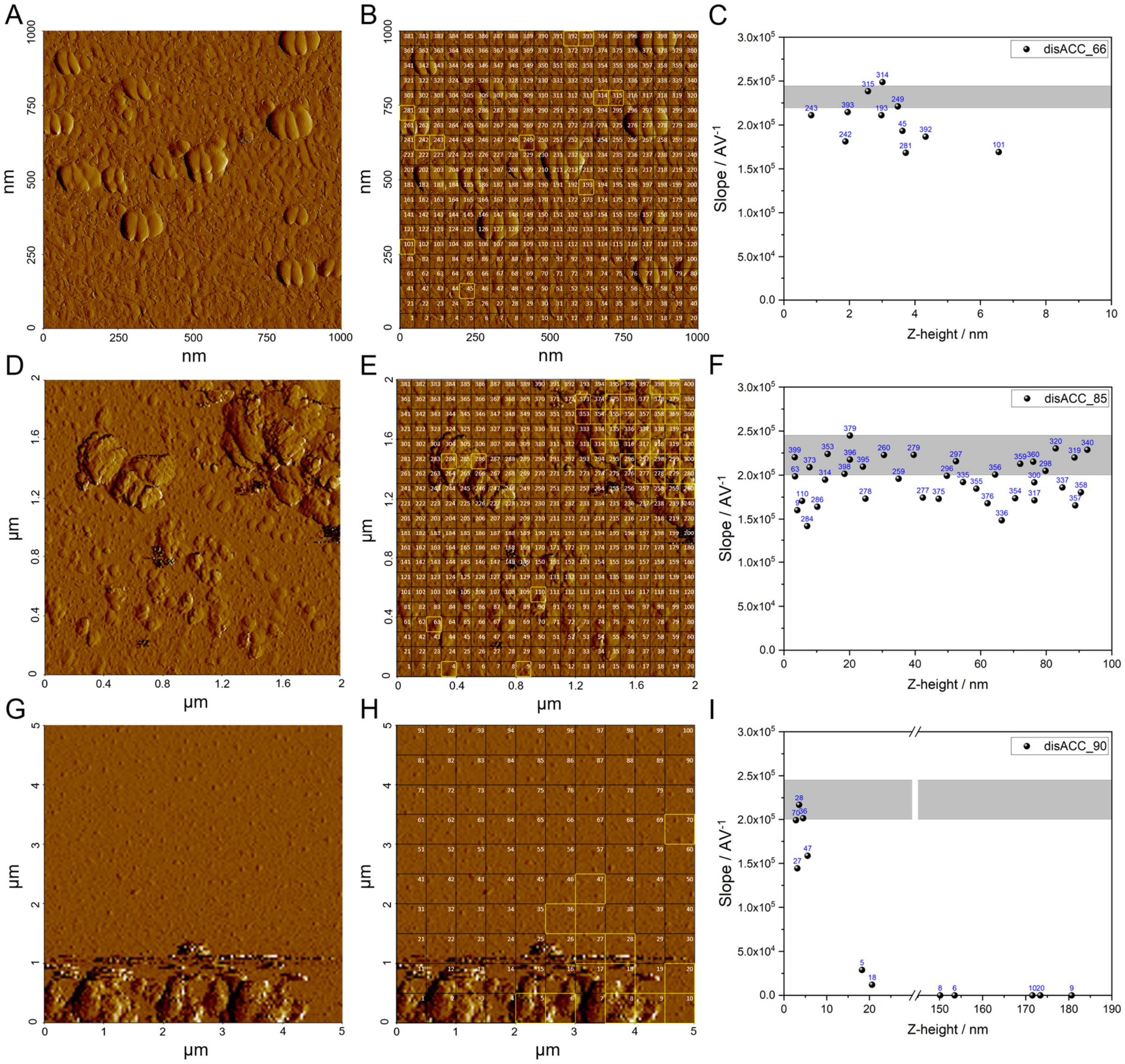

Supplementary Figure 23. C-AFM measurement of polymer-free ACC particles. The conductivity of ACC nanoparticles (preparation see methods section in the main manuscript) was determined. a) NCM amplitude map of a measurement of ACC nanoparticles distributed on the substrate. The particles on the wafer show a diameter of 20-50 nm. b) Amplitude map overlayed with the grid of points measured in spectroscopy mode. In yellow, the data points considered for evaluation are highlighted. c) Plot of measured slope of the I/V diagram for each selected data point in dependence of the starting Z-height of the spectroscopy measurement. The area highlighted in gray corresponds to the average value determined for measurements on the Au substrate (standard deviation of at least 30 evaluated points), indicating the maximum conductivity that can be determined. Interestingly, although the particles show a diameter of 20-50 nm, the Z-height detected during measurement was less than 5 nm for most measurements, showing that the particles are deformed when precipitated on a wafer due to their gel-like properties, as detected for similarly prepared samples before (see also Supplementary Figure 2).[21] This is also evident by the anisotropic, elongated shape of the particles due to the forces present during spin-coating the particles. In addition, it is possible that the AFM tip is penetrating into the particles when approaching for C-AFM spectroscopy measurements. Due to the high voltage applied (up to 2 V) we cannot exclude leaking voltage at Z-heights below 20 nm, especially considering the results for non-conductive vaterite reference nanoparticles (Supplementary Figure 21f). We therefore decide not to comment on measurements with Z-heights below 20 nm. d-f) Corresponding

measurements for a different area of the ACC sample, this time showing presence of larger particles. Interestingly, the ACC structures show good conductivity across all measured points, similar to the conductive Au reference sample (Supplementary Figure 21c). g-i) Corresponding measurements for another area on the sample, showing the presence of even lager structures, with Z-heights above 100 nm. This time, the conductivity rapidly decreases for increasing Z-heights, and already at 20 nm, there is no conductivity detected. This is in contrast to the measurements discussed before (in d-f). In fact, for all investigated areas of the sample, one of the two different trends was visible. Either high conductivity up to a Z-height of 100 nm or rapid decrease to 0 within a Z-height of 20 nm. We attribute this to different types of ACC structures present. If only a loose aggregation of nanoparticles is present, there is no conductivity detected, as several nanoparticles are present between the AFM tip and the substrate and conductivity on the surface of the particles is very low, especially if the particles are not in proper contact. That the particles are in loose contact is also visible from the "glitches" in the amplitude map (g), indicating movement of particles during NCM measurement. The other case is the presence of large, continuous ACC structures. These could be either formed by dehydration of a large-scale liquid mineral phase, as detected for polymer free ACCs before,[21] or by the fusion of liquid-like (viscoelastic) ACC particles.[23] In either case, a continuous ACC structure will have much better contact with the wafer without any significant interruption of the bulk phase across the structure, resulting in better conductivities.

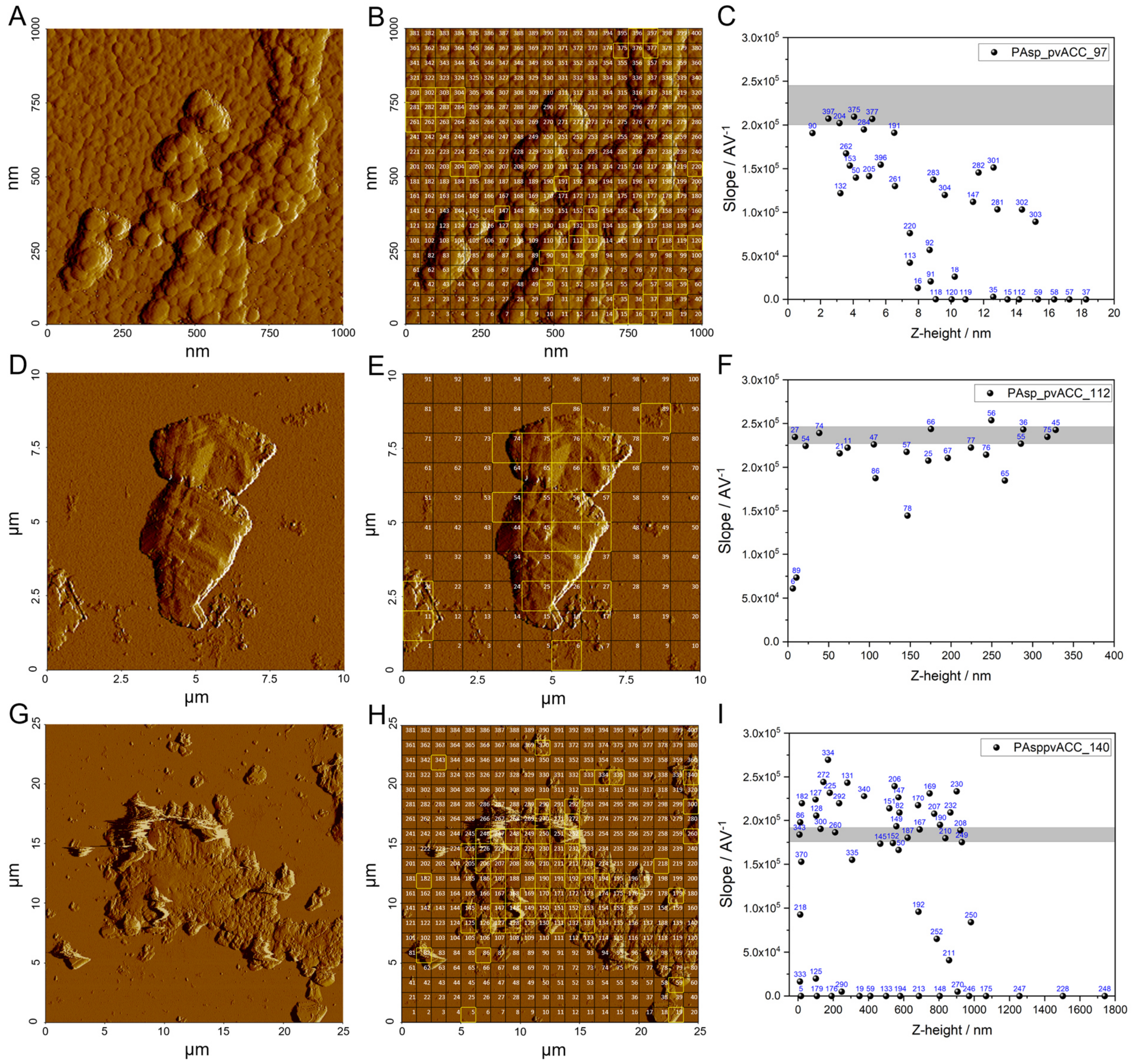

Supplementary Figure 24. C-AFM measurement of polymer-stabilized ACC (PAsp_ACC) particles. The conductivity of the PAsp stabilized proto-structured ACC particles (PAsp_ACC, preparation see methods section in the main manuscript) was determined. a) NCM amplitude map of a measurement of PAsp_ACC nanoparticles distributed on the wafer. b) Amplitude map overlayed with the grid of points measured in spectroscopy mode. In yellow, the data points considered for evaluation are highlighted. c) Plot of measured slope of the I/V diagram for each selected data point in dependence of the starting Z-height of the spectroscopy measurement. The area highlighted in gray corresponds to the average value determined for measurements on the Au substrate (standard deviation of at least 30 evaluated points), indicating the maximum conductivity that can be determined. As discussed in Supplementary Figure 23, below 20 nm leakage current likely plays a role, so it cannot be commented on the conductivity of individual PAsp_ACC nanoparticles. d-f) Corresponding measurements for a large ACC structure. These structures are the remaining of the µm-size polymer-stabilized liquid precursor droplets, that were causing the decrease in transmission in the titration experiments (see Supplementary Figure 35). Surprisingly, these structures survived the quenching procedure. The ACC shows good conductivity for Z-heights over 300 nm. g-i) Corresponding measurements for an even larger PAsp_ACC structures showing good conductivity across a Z-height of almost 1 µm, demonstrating that PAsp_ACC shows conductivity on a scale well beyond nm range.

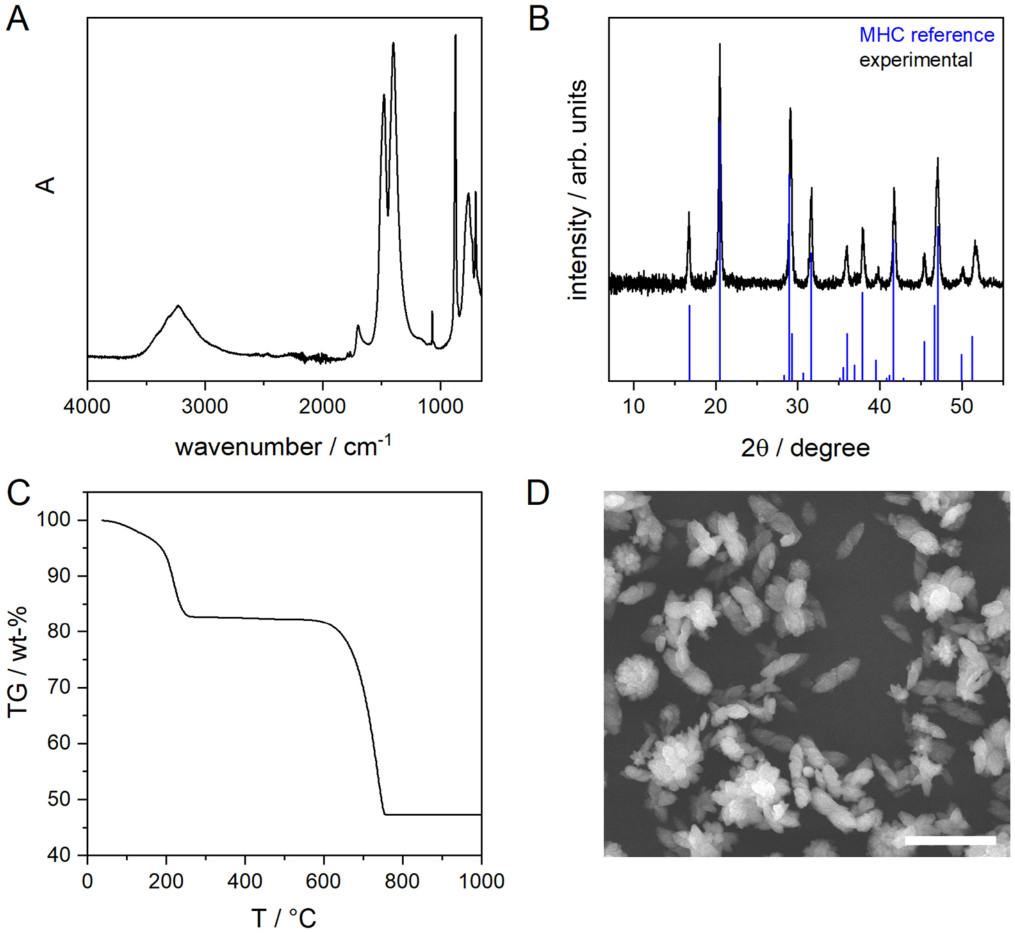

Supplementary Figure 25. Characterization of the synthesized Monohydrocalcite (MHC) sample. a) ATR-FTIR spectroscopy, b) PXRD with literature reference data,[24] c) TGA and DSC analysis and d) SEM characterization. The data confirms nicely to values reported in literature[25] and no indications for presence of calcite, vaterite, aragonite or ACC were visible.

2. Calculation of bicarbonate binding

2.1. Calculation of free ion products

2.1.1. Calibration of Ca-ISE

For evaluation of the calibration of the Ca-ISE, $\ln(a_{free}(Ca^{2+}))$ was plotted against $U(Ca^{2+})$. Then, the relevant potential regime for each measurement (e.g., from -50 to -10 mV) was fitted linearly. The experimentally measured potential can then be used to calculate the calcium activity according to the Nernst equation:[26]

$$a_{\text{free}}(\text{Ca}^{2+}) = \exp\left(\frac{2F(U_0 - U(\text{Ca}^{2+}))}{\text{R}T}\right) \tag{1}$$

2.1.2. Calculation of free ion products

In the following calculations, solutions were treated ideally, which is an acceptable assumption for the investigated concentration range, as demonstrated before.[26] To calculate the free ion product (corresponding to the ion activity product (IAP) if solutions are treated ideally), the concentration of free Ca^{2+} (determined from the Ca-ISE) was multiplied with the concentration of free carbonate ions.[27] The free carbonate concentration was calculated using a 1:1 binding ratio of calcium and carbonate according the following equation

$$\begin{aligned} c_{\text{free}}(\text{CO}_3^{2-}) = {} & \frac{1}{V_{\text{total}}}\left(n_{\text{total}}(\text{carb}) - \left(n_{\text{added}}(\text{Ca}^{2+}) - n_{\text{free}}(\text{Ca}^{2+})\right)\right) \\ & \cdot\left(\left(\frac{10^{-pH}}{10^{-10.33}}\right) + \left(\frac{(10^{-pH})^2}{10^{-6.35}\cdot 10^{-10.33}}\right) + 1\right)^{-1} \end{aligned} \tag{2}$$

Where $n_{total}(carb)$ describes the total amount of carbonate species in the system (10 mM for standard experiments). Dilution effects during the experiments, e.g., during adjustment of initial pH value of the solution, were considered for the calculations.

2.2. Calculation of microscopic binding parameters of PNC association

Microscopic binding parameters of the multiple binding model used to describe PNC equilibria were calculated as demonstrated in the original work of Gebauer et. al.[27] Thereby, the microscopic number of calcium ions that bind a carbonate ion, x, and the microscopic binding equilibrium constant, K, are determined. For each measurement the $n_{free}(CO_3^{2-})$, $n_{bound}(Ca^{2+})$ and $c_{free}(Ca^{2+})$ data from the titration was fitted linearly in the prenucleation regime. The linear fits of $n_{bound}(Ca^{2+})$ and $c_{free}(Ca^{2+})$ were forced to intersect y = 0. This is necessary, because the electrode has a nonlinear behavior close to $c_{free}(Ca^{2+}) = 0$, which causes unrealistic values in this region, which in turn will result in a large error during the reciprocal plotting of the data in following steps of the evaluation procedure (eq. 3). The binding parameters x and K can then be calculated using a linear fit of the following equation:

$$1+\left(\frac{n_{\mathrm{free}}(\mathrm{CO}_3^{2-})}{n_{\mathrm{bound}}(\mathrm{Ca}^{2+})}\right)=\frac{1}{x}\cdot\frac{1}{x\cdot K}\cdot\frac{1}{c_{\mathrm{free}}(\mathrm{Ca}^{2+})} \quad (3)$$

This procedure was repeated for each titration measurement. At least 3 independent titration experiments were evaluated.

In case of experiments with polymers, the same method can be used, as the polymer concentrations used were very low (10 mg/L), and the error caused by calcium adsorption by the carboxyl groups of the polymer (Langmuir binding isotherm)[28] is within the experimental error of the experiment, as discussed in the main text.

2.3. Calculation of bicarbonate binding

2.3.1. Experimental strategy

To calculate the amount of bound bicarbonate in the prenucleation regime, the amount of bound CO_3^{2-} determined from the Ca-ISE (by calculating Ca^{2+} binding and assuming a 1:1 binding ratio of bound CO_3^{2-} and bound Ca^{2+}) is compared with the amount of CO_3^{2-} binding "visible" to the pH electrode due to changes in the buffer equilibrium (Supplementary Figure 26). Essentially, if bicarbonate binding takes place in addition to carbonate binding, the buffer equilibrium is shifted less and the change in pH value upon calcium addition/(bi)carbonate binding is reduced. Quantitative evaluation of these effects allows determining the amount of bicarbonate binding in the prenucleation regime.

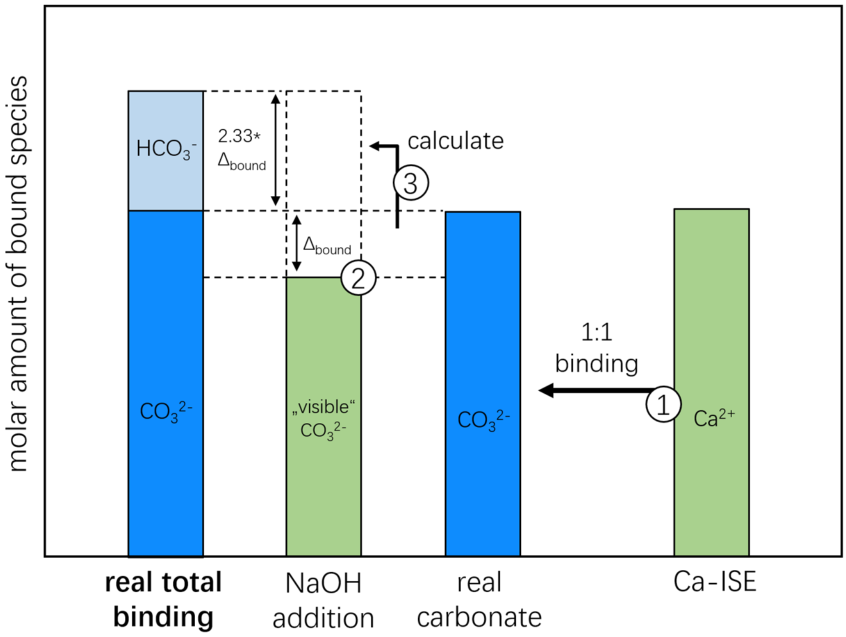

Supplementary Figure 26. Strategy to determine bicarbonate binding. Molar amounts of bound species are shown and whether they can be determined directly from experimental measurements (green) or accessed from calculations (blue). In titration experiments, the amount of bound calcium can be determined directly from the measured calcium potentials from the Ca-ISE. Assuming a 1:1

binding ratio of CO_3^{2-} and Ca^{2+} (refer to following text for justification of this assumption), the amount of bound CO_3^{2-} can be calculated from the amount of bound Ca^{2+} (1). Upon binding of CO_3^{2-}, the carbonate is removed from the buffer equilibrium ($HCO_3^- \rightleftharpoons H^+ + CO_3^{2-}$), resulting in the release of H^+, that are compensated by automatic addition of NaOH. Therefore, from the amount of added NaOH in the titration, the removal of CO_3^{2-} from the equilibrium can be quantified. As described in the main text (Figure 1c in the main text), there is a significant difference (Δ_{bound}) between this calculated value and the binding determined from calcium binding (2). The explanation for this difference is simultaneous binding of HCO_3^-, causing a reduced H^+ release if CO_3^{2-} is removed from the equilibrium upon binding to calcium. Quantitative evaluation shows, that 2.33 mol HCO_3^- must be bound to make 1 mol CO_3^{2-} "invisible" to the pH electrode. Using this value, the real binding speciation can be calculated (3).

2.3.2. Determination of bound carbonate via bound Ca^{2+}

Due to the difference in activity coefficients of HCO_3^- and CO_3^{2-} involved in the calculations, it must be worked with actual concentrations, i.e., the solutions cannot be treated as ideal solutions. The free calcium concentration is accessible from the Ca-ISE, if the calibration is performed in ionic strength adjusted solutions.[26] The starting ionic strength of the regular experiments with 10 mM carbonate buffer at pH 9.8 was calculated using the Davies equation. The determined ionic strength of the solution is 14.5 mM, with the value only changing little during the first few hours of the experiments.

The ionic strength adjusted calibration of the Ca-ISE was carried out by dosing 20 mM $CaCl_2$ solution with 0.01 mL/min into 20 mL of 14.5 mM NaCl solution (see methods section in the main manuscript). In this way, the activity coefficient for Ca^{2+} ions was experimentally determined as $y(Ca^{2+}) = 0.63$ in the first 2 hours of each experiment, which is in good agreement with values determined in similar conditions.[26,29] The free calcium concentration can then be calculated from the ion activities measured by the Ca-ISE according to:

$$c_{\text{free}}(\text{Ca}^{2+}) = \frac{a_{\text{free}}(\text{Ca}^{2+}) \cdot c^0}{y(\text{Ca}^{2+})} \qquad (4)$$

According to earlier experiments,[27] a 1:1 binding ratio of calcium and carbonate in the prenucleation regime was determined for additive-free systems within experimental accuracy, while no bicarbonate association was detected. This can be explained by the 2 orders of magnitude smaller binding constant of $Ca\text{-}HCO_3^+$ (20 M^{-1}) compared to $Ca\text{-}CO_3^0$ (1700 M^{-1}).[15] The calcium-bicarbonate association only becomes relevant at pH values below pH 9.0 due to the low amount of carbonate present in the buffer equilibrium in this pH range. Our experiments are performed at pH 9.8, and using the ion association constants, a value of 4.1% ± 4.4% of bicarbonate binding to calcium can be calculated, which is within the experimental accuracy of a 1:1 binding ratio determined via NaOH addition in earlier experiments[27] (see Figure 1c and d in the main text, data for reference experiments). Therefore, in our experiments, assuming a 1:1 binding ratio of calcium and carbonate in the prenucleation regime for additive-free systems is justified. The amount of bound carbonate according to the Ca-ISE can be calculated according to

$$n_{\mathrm{bound,ISE}}(\mathrm{CO_3^{2-}}) = n_{\mathrm{bound}}(\mathrm{Ca^{2+}}) = n_{\mathrm{total}}(\mathrm{Ca^{2+}}) - n_{\mathrm{free}}(\mathrm{Ca^{2+}}) \quad (5)$$

With $n_{total}(Ca^{2+})$ being accessible via the known values of calcium concentration and addition rate and $n_{free}(Ca^{2+})$ being accessible from the detected concentrations by the Ca-ISE and the total volume of the solution. Dilution effects of the addition of $CaCl_2$ and NaOH solutions need to be considered for the evaluations.

These calculations are straightforward for additive-free systems, however, in presence of polymer additives, the binding of Ca^{2+} to carboxyl groups needs to be considered, as this will affect the 1:1 binding assumption. One possibility is the determination and subtraction of the binding isotherm,[28] however due to potentially coupled equilibria of free Ca^{2+} ions that are binding to carbonate and polymer at the same time, it is very difficult to isolate the two binding processes. Therefore, only the later stages of the titration experiment are considered, at which the initial (Langmuir) binding of Ca^{2+} to the carboxyl groups is not relevant (Supplementary Figure 27). In addition, only the slope of the titration curve was considered, thereby eliminating the effect from Ca^{2+} binding to carboxyl groups on the total amount of bound Ca^{2+} species.

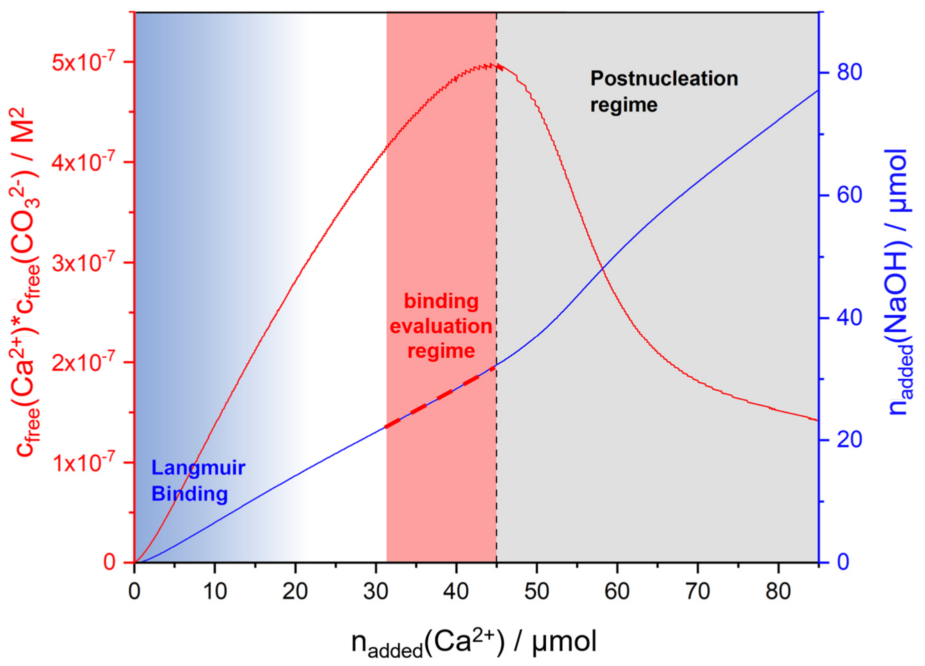

Supplementary Figure 27. Relevant regime in titration experiments to determine bicarbonate binding. In the beginning of the experiments, Ca^{2+} ions are bound in PNCs as well as to the polymer carboxyl groups. The binding to the polymer groups cannot be unambiguously isolated in the calculations, therefore the beginning of the titration (area highlighted in blue) was not considered for evaluation. In the later stages of the experiment, nucleation of solids takes place (highlighted in gray). Therefore, only the late part of the prenucleation regime (highlighted in red) was considered for binding evaluation. Calculations were performed using the slope of NaOH addition and Ca^{2+} binding, respectively.

2.3.3. Determination of "visible" bound carbonate via NaOH addition

It is possible to determine the amount of bound CO_3^{2-} ions from the addition of NaOH in pH-constant-experiments, as the carbonate buffer equilibrium is shifted upon removal of carbonate ions. However, the quantitative evaluation of NaOH data is quite challenging, as already small changes in the environment, e.g., caused by CO_2 in-diffusion into the NaOH solution, can introduce a large error into the measurements. Indeed, while determinations using NaOH addition rates have been performed before,[27] there have also been reports claiming that determinations using amounts of added NaOH are not sufficiently accurate to quantitatively determine binding speciations.[29] In the following, a strategy for quantitative evaluation of experiments involving NaOH addition is presented. The strategy involves evaluation of the NaOH addition from calibration experiments (dosing $CaCl_2$ into water) and polymer-free reference experiments (dosing $CaCl_2$ into carbonate buffer) to extract parameters to correct experiments performed in presence of polymers. In the first step, the slope of added NaOH in calibration experiments (Supplementary Figure 28a, black curve) was subtracted from the NaOH addition for reference experiments (Supplementary Figure 28a, red curve). In this way, the amount of NaOH added to neutralize the added $CaCl_2$ solution is subtracted, leaving only NaOH addition due to changes in carbonate buffer equilibrium. From the resulting data after subtraction (Supplementary Figure 28b), the postnucleation slope (molar amount added NaOH per added molar amount of $CaCl_2$) can be evaluated. In the postnucleation regime for reference experiments, all added calcium ions bind to carbonate ions (particle growth) and 1:1 binding takes place. Therefore, the determined slope can be corrected by multiplying the whole curve with a "correction factor", so the post-nucleation slope equals the theoretical value of 0.7 mol NaOH added per mol bound CO_3^{2-} (see next paragraph for calculation). This "correction factor" accounts for all effects that lead to a "nonideal" behavior of the NaOH titration in our experiments, for example:

- neutralization of CO_2 present in the experiment, e.g., due to in-diffusion
- measurement error in preparation of NaOH solution
- temperature differences
- changes in ionic strength

Using this "internal" calibration for calculations involving NaOH addition, we can improve the data quality significantly. From the corrected NaOH additions (Supplementary Figure 28c) we can now determine the slope of added NaOH in the prenucleation regime and calculate the amount of bound CO_3^{2-}, that is "visible" to the pH electrode.

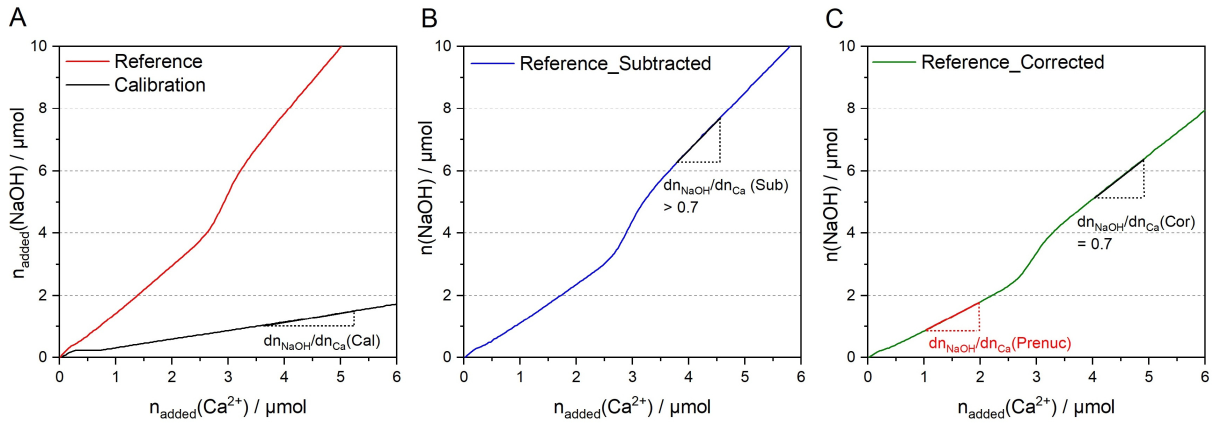

Supplementary Figure 28. Strategy for determinations using NaOH addition. a) From a polymer-free reference experiment (red), the slope of the calcium calibration experiment (black) is subtracted to yield b) a corrected reference curve (blue). This curve can be multiplied by a "correction factor" to adjust the postnucleation slope to the theoretical value. c) From This corrected curve (green), the prenucleation slope (red slope) can be evaluated to determine prenucleation binding characteristics.

For experiments with polymer, due to interactions of the polymer with the formed $CaCO_3$ particles in the postnucleation regime, as discussed in the main text, it is not possible to determine the "internal" correction factor from the postnucleation slope of the same experiment. Therefore, the slope factor from at least 6 reference experiments was averaged and used for correction of the polymer experiments. All conditions (especially the used NaOH solutions) were kept constant during performance of reference and polymer experiments. As described earlier, only the middle part of the titration curves was used for evaluation (Supplementary Figure 27), and the same part of the curves was used as for the determinations from the Ca-ISE. As result, a slope (added amount of NaOH per added amount of $CaCl_2$) in the prenucleation regime is obtained, that can be converted to the amount of (apparently) bound carbonate, as described in the following paragraph.

2.3.4. Determination of amount of bound bicarbonate

As shown in Supplementary Figure 26, for polymer experiments the values of CO_3^{2-} binding determined by the Ca-ISE and determined via the NaOH addition are different. This difference Δ_{bound} can be calculated according to:

$$\Delta_{\mathrm{bound}} = n_{\mathrm{bound,ISE}}(\mathrm{CO_3^{2-}}) - n_{\mathrm{bound,NaOH}}(\mathrm{CO_3^{2-}}) \qquad (6)$$

The Δ_{bound} can be used to calculate the amount of bicarbonate binding in the system. Therefore, it needs to be determined, how much H^+ is released upon shifting the equilibrium, if one CO_3^{2-} ion is removed from the buffer equilibrium and how much H^+ is consumed, if one HCO_3^- is removed from the equilibrium. The removal of one carbonate ion (in terms of total concentrations) from the buffer equilibrium causes the relative ratio of carbonate and bicarbonate ions to change, i.e., a perturbation is caused (Supplementary Figure 29a). As the pH is held constant, the equilibrium readjusts so the activity ratio stays constant. Note that

the ratio is in terms of activities and can be calculated using the Henderson-Hasselbalch equation (determination of the activity ratio) and the Davies equation (determination of actual activities).

a Carbonate binding

	c / mM	a / mM
CO_3^{2-}	2.98	1.83
HCO_3^-	7.02	6.22
	10 total	ratio 3.39

binding →

	c / mM	a / mM
CO_3^{2-}	1.98	1.22
HCO_3^-	7.02	6.22
	9 total	ratio 5.09

readjust →

	c / mM	a / mM
CO_3^{2-}	2.68	1.65
HCO_3^-	6.32	5.60
	9 total	ratio 3.39

b Bicarbonate binding

	c / mM	a / mM
CO_3^{2-}	2.98	1.83
HCO_3^-	7.02	6.22
	10 total	ratio 3.39

binding →

	c / mM	a / mM
CO_3^{2-}	2.98	1.83
HCO_3^-	6.02	5.33
	9 total	ratio 5.09

readjust →

	c / mM	a / mM
CO_3^{2-}	2.68	1.65
HCO_3^-	6.32	5.60
	9 total	ratio 3.39

Supplementary Figure 29. Changes in carbonate buffer equilibria. a) 1 mM Carbonate is removed from a 10 mM carbonate buffer at pH 9.8. The equilibrium readjusts resulting in the release of 0.7 H^+. b) 1 mM bicarbonate is removed from a 10 mM carbonate buffer at pH 9.8. The equilibrium readjusts resulting in the consumption of 0.3 H^+.

It is evident that 0.7 mM bicarbonate transform to 0.7 mM carbonate and 0.7 mM H^+, if 1 mM carbonate is removed from the buffer equilibrium (Supplementary Figure 29a). In the same way, it can be calculated that the removal of 1 mM bicarbonate results in the consumption of 0.3 mM H^+ (Supplementary Figure 29b).

Therefore, 0.7 mM/0.3 mM = 2.33 times more protons are generated for the binding of one carbonate than are consumed during the binding of one bicarbonate ion. This means that it is necessary to bind 2.33 bicarbonate ions to make the binding of one carbonate ion "invisible" to the pH electrode. From this relation, we can use Δ_{bound} to determine the bicarbonate binding according to

$$n_{\text{bound}}(\text{HCO}_3^-) = 2.33 \cdot \Delta_{\text{bound}} \tag{7}$$

with $n_{\text{bound}}(HCO_3^-)$ describing the bound bicarbonate ions in the system in the prenucleation regime. In this way, the relative amount of bound bicarbonate from the total amount of bound species can be calculated according to

$$n_{\text{bound}}(\text{HCO}_3^-) = \frac{2.33 \cdot \Delta_{\text{bound}}}{3.33 \cdot \Delta_{\text{bound}} + n_{\text{bound,NaOH}}(\text{CO}_3^{2-})} \tag{8}$$

As only the slopes of the titration data are evaluated, the quantities stated in eq. 8 are usually per added amount of Ca^{2+}.

3. Discussion of titration experiments and additive-controlled mineralization

3.1. Basic titration experiments

In potentiometric titration experiments, dilute $CaCl_2$ solution is slowly dosed into a stirred solution of carbonate buffer and polymer, while the pH is kept constant by automatic addition of NaOH. During the process, parameters are recorded in-situ using potentiometric electrodes, allowing to gain insights into the entire mineralization process, starting from the undersaturated solution up to the final crystalline particles. In addition, mineral samples can be isolated at distinct stages in the pathway and analyzed using ex-situ techniques, such as Fourier-transform infrared spectroscopy (FTIR) and scanning electron microscopy (SEM). Using this approach and well-defined polycarboxylate additives, i.e., polymers with small polydispersities and comparable chain lengths, the effect of polymers on the distinct stages along the $CaCO_3$ formation pathway can be systematically investigated.

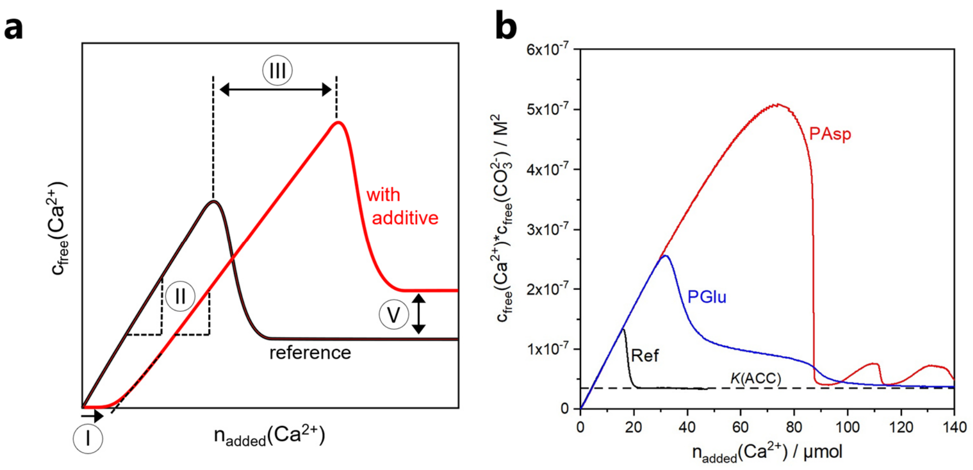

Supplementary Figure 30. Illustration of potentiometric titration experiments in presence of polymer additives. A) Schematic depiction of the effect of different additive interactions on titration curves. Experiments are always considered in regard to the additive-free reference. Please refer to the text for explanations. B) Development of free ion product during titration experiments without additive (black) and in presence of 10 mg/L PAsp (red) and PGlu (blue). Experiments were performed by dosing 20 mM $CaCl_2$ solution into 50 mL of 10 mM carbonate buffer at pH 9.4. The dotted line represents the solubility product of ACC formed after nucleation in the reference experiment. The data is exemplary shown for pH 9.4, for the full set of experimental data see Supplementary Figure 33.

In titration experiments, the development of free calcium concentration is recorded by a calcium ion selective electrode (ISE) during addition of $CaCl_2$ solution into a polymer/carbonate solution. Then, the data recorded with additives in the carbonate buffer solution is compared to a polymer-free reference experiment, allowing the identification of different types of interactions (Supplementary Figure 30a). The most pronounced effects are the change in offset of the curve, indicating calcium ion binding by additives (type I),[28] changes in the slope of the curve in the prenucleation regime, indicating interactions with prenucleation clusters (PNC) (type II),[27] shift in the maximum of the curve, indicating

nucleation inhibition (type III) and change in the plateau of free calcium concentration detected after the drop, indicating the presence of different species in the post-nucleation regime (type V). Analyzing the titration curves in this regard for poly(aspartic acid) and poly(glutamic acid) with a polymerization degree of 50 (PAsp and PGlu) (Supplementary Figure 30b), it is evident, that no calcium binding (type I) can be detected, due to the low additive concentrations used in the experiments (10 mg/L). However, although concentrations are very low, a strong inhibition of nucleation is detected (type III), with PAsp showing a stronger inhibition compared to PGlu, as reported before.[30-32] Interestingly, no effect on PNC equilibria could be detected (type II), at least for the investigated concentrations, showing that the strong nucleation inhibition likely cannot be explained by (de)stabilization of PNCs or effects on PNC equilibria. After the maximum in the curves, the polymers also show a different behavior, with a phase of higher solubility product formed in presence of PGlu (type V), while in case of PAsp, a product is formed with similar solubility as in the reference experiments, which, according to the solubility product, corresponds to amorphous calcium carbonate (ACC) formed at the respective pH value.[27,33] Although there is no effect on the solubility product of the initially formed phase detected, in case of PAsp the free calcium concentration increases again before additional drops are detected. This indicates, that the particle growth is inhibited after nucleation, presumably by adsorption of PAsp on the formed particles.[32]

3.2. Characterization of isolated ACC samples

To investigate the species present at distinct points in the titration experiment, samples were isolated along the titration curve and characterized using FTIR spectroscopy (Supplementary Figure 31) and SEM (Supplementary Figure 32). Prior to the maximum in the titration curves, ACC particles are present with a size of less than 100 nm. The ACC particles then transform to crystalline $CaCO_3$ upon drop in calcium potential. In additive free systems, this takes place by a dissolution-recrystallization process.[21] In all experiments, the first detected crystalline phase is detected at the maximum in the titration curve in the form of spherical vaterite particles (several 100s of nm in size). Without additives, these particles then transform to larger hexagonal vaterite crystals, which in turn (slowly) transform into the thermodynamically stable $CaCO_3$ polymorph calcite. In case of experiments with polymers, this transformation to calcite is not detected and the small spherical vaterite particles are stabilized, presumably by adsorption of the polymer onto the particles, as seen in the PAsp titration curves. In case of PGlu, on the plateau with higher solubility after the maximum, there are small nanoparticles visible, that can be identified as ACC according to FTIR (Supplementary Figure 31f), showing the stabilizing effect of PGlu on ACC. This is reminiscent of biomineralization, as glutamic acid rich peptides are often associated with ACC, while peptides rich in aspartic acid are upregulated during formation of crystalline $CaCO_3$[34] It is intriguing, that PGlu seems to show a strong stabilizing effect on ACCs, that remain stable even in presence of crystalline particles, while the nucleation inhibition, and therefore stabilization of liquid and amorphous precursor species in the prenucleation stage, is much stronger for PAsp.

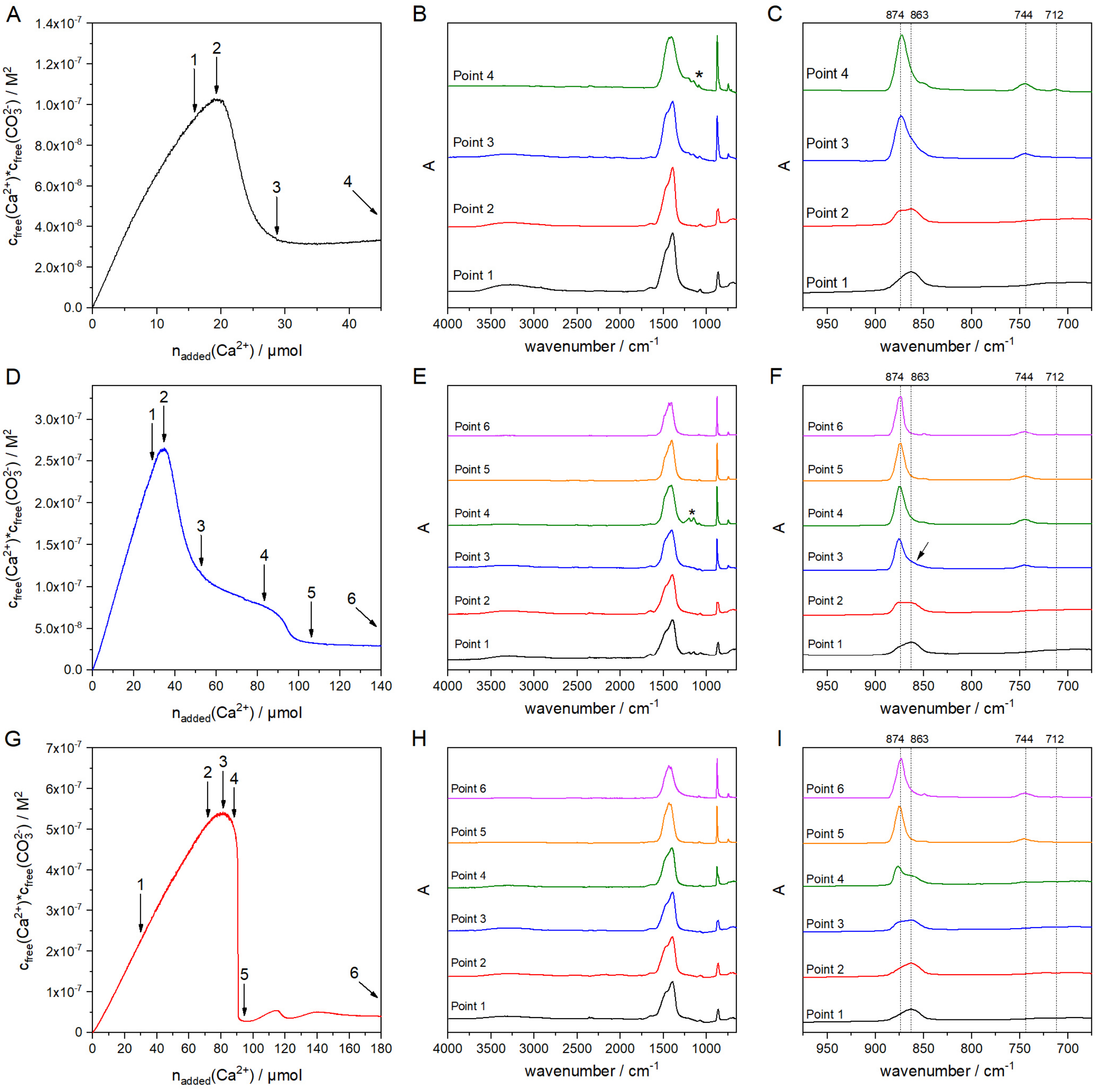

Supplementary Figure 31. Isolation of samples at different stages in the titration. Titration experiments were performed at pH 9.4, using polymer concentrations of 10 mg/L. a) Reference titration curve. Arrows indicate points, at which samples were isolated (see methods section in the main manuscript). The last isolation (Ref: point 4; PAsp/PGlu: point 6) was performed 16 h after the nucleation point, corresponding to the addition of additional 400 µmol Ca^{2+}. b) ATR-FTIR spectra for the isolated samples. The asterisks indicate a small contribution from PTFE that was rubbed off from the PTFE stir bar. c) Magnification of the FTIR spectra in the range from 650 to 1000 cm^{-1}. The ν_2 carbonate vibration can be used to distinguish crystalline (sharp band at 874 cm^{-1}) from amorphous $CaCO_3$ (broad band at 863 cm^{-1}). The crystalline polymorphs can further be distinguished by the vibrational bands at 712 cm^{-1} (calcite) and 744 cm^{-1} (vaterite).[8] The same analysis was performed for d-f) PGlu and g-i) PAsp. For all samples, in the prenucleation regime, i.e., prior to the maximum in free ion product, ACC is present, that transforms to vaterite upon drop in the free ion product. An interesting feature is the shoulder in the experiments with PGlu (point 3 and 4 in d). IR characterization shows a visible shoulder from ACC for this sample (arrow in f), confirming that significant amounts of amorphous mineral are present at this point. This shoulder has disappeared at point 5, in correspondence with a drop in free ion product in the titration (d), therefore, the phase with high solubility in titration experiments can be attributed to a (polymer stabilized) amorphous phase.

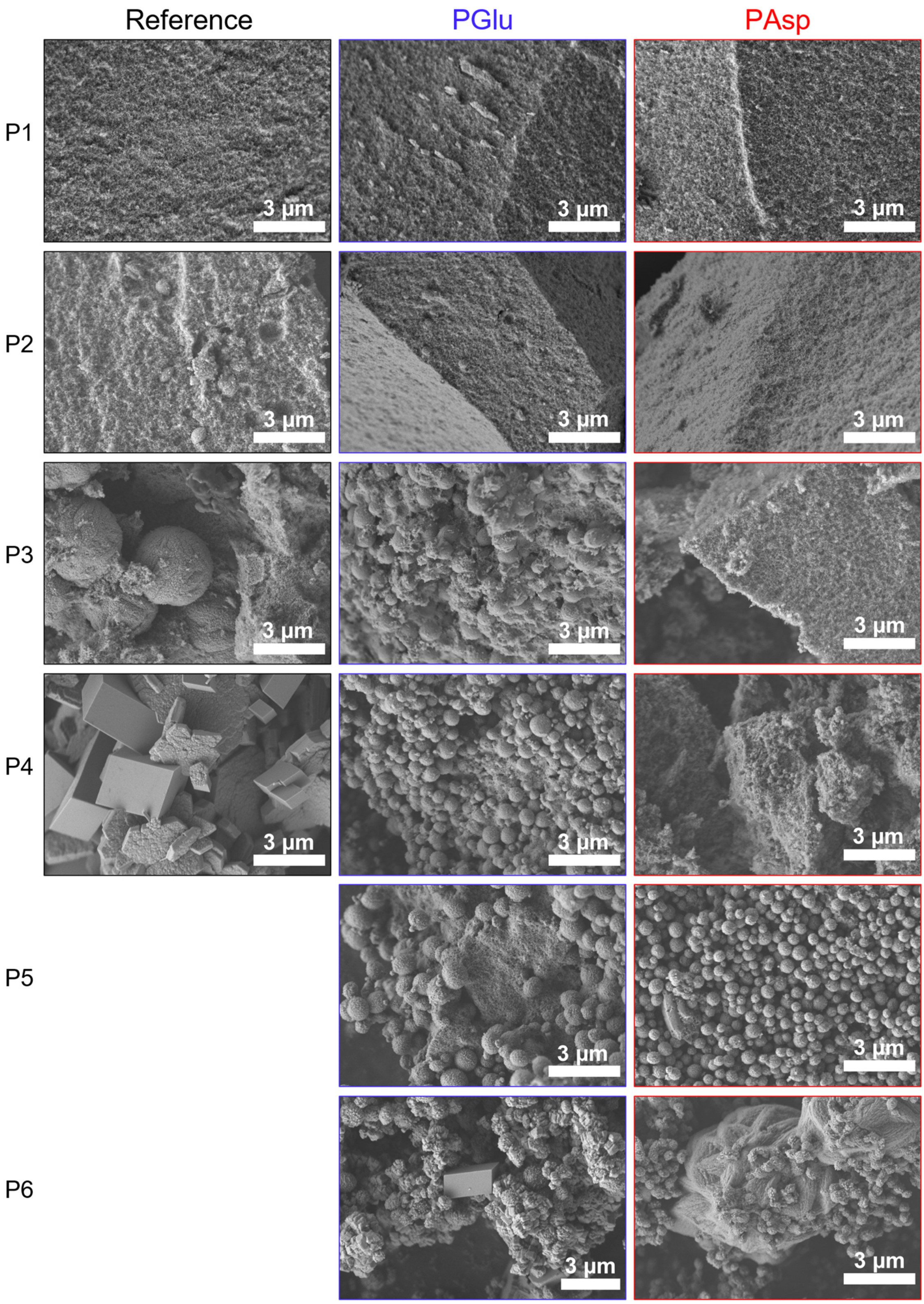

Supplementary Figure 32. SEM investigations of isolated samples from titration experiments. Samples were isolated as shown in Supplementary Figure 31. In the beginning of the titration experiments, ACC nanoparticles are visible that transform to spherical vaterite particles upon crystallization. For the samples isolated 16 h after nucleation, larger crystalline structures are visible, with drastic changes of particle morphology visible in presence of polymers. For PGlu, small nanoparticles are also visible after nucleation (P3-P5), likely corresponding to the stabilized ACC phase detected by ATR-FTIR (Supplementary Figure 31f, arrow).

Titration experiments at different pH values (Supplementary Figure 33) reveal that at all investigated pH values the previously described trends are visible, with PAsp showing stronger crystallization inhibition, while PGlu possesses a stronger post-nucleation ACC stabilization. Interestingly, this kinetic stabilization of ACC particles is stronger at higher pH values, visible by the plateau with higher solubility being present for a longer time. Even for PAsp, at high pH (pH 10.2, Supplementary Figure 33d) a plateau becomes visible while at lower pH values (pH 9.0, Supplementary Figure 33a), the plateau for PGlu has almost disappeared. This effect could be caused by a dependence of stabilizing efficacy on ACC proto-structure.[8] Experiments performed with poly(acrylic acid) (PAA), reveal the same type of interactions, while they show the strongest inhibition of all investigated polymers (Supplementary Figure 34). Although our further results focus on elucidating effects for PAsp and PGlu due to their relevance in biomineralization, we argue that the essential conclusions will be valid for PAA as well due to the similarity in the titration experiments.

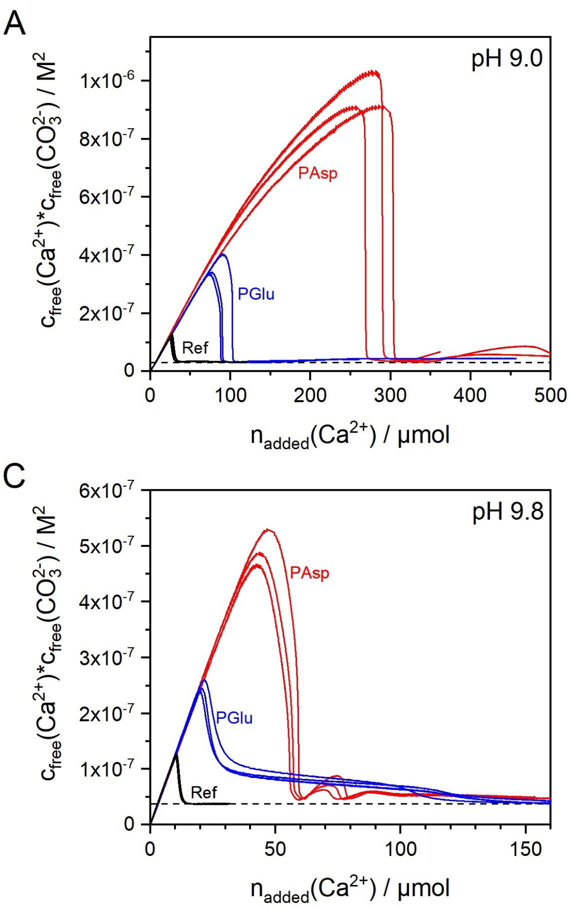

Supplementary Figure 33. Titration experiments performed at different pH values. Experiments were performed by slowly dosing 20 mM $CaCl_2$ into a solution of 10 mM carbonate buffer and 10 mg/L polycarboxylate additive (PAsp – red; PGlu – blue). In addition, the reference experiment in absence of polymer (black) is shown. The experiments showed a good repeatability across the investigated pH values of a) pH 9.0 b) pH 9.4 c) pH 9.8 and d) pH 10.2. The dotted line indicates the solubility product of the ACC phase formed at each pH (pH 9.0/9.4: proto-calcite ACC; pH 9.8/10.2: proto-vaterite ACC).[27] Experimental details for titration experiments are described in the methods section in the main manuscript.

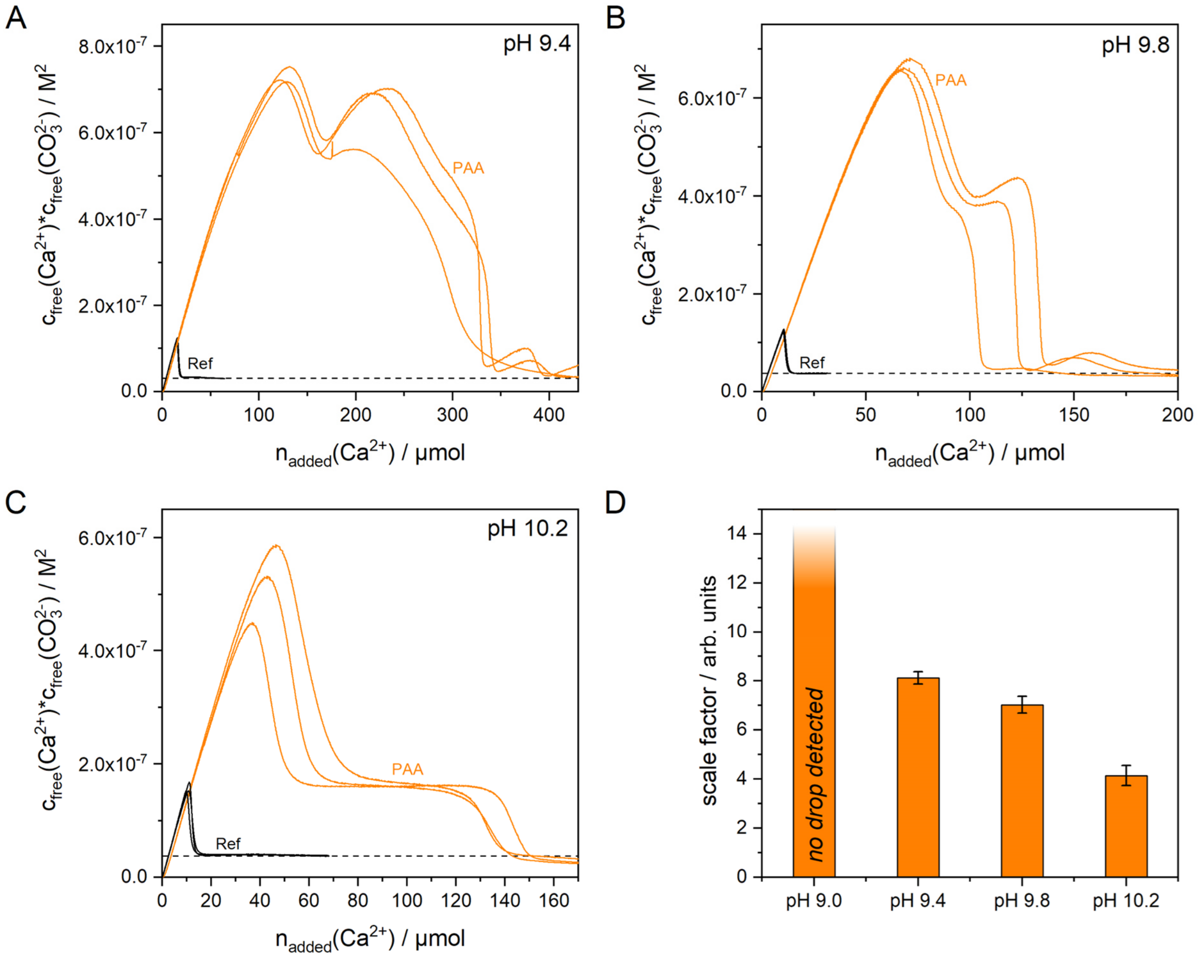

Supplementary Figure 34. Titration experiments with poly(acrylic acid). Experiments were performed by slowly dosing 20 mM $CaCl_2$ into a solution of 10 mM carbonate buffer and 10 mg/L poly(acrylic acid) (PAA, orange). The reference experiment in absence of polymer (black) is shown as well. The experiments showed a good repeatability across the investigated pH values of a) pH 9.4 b) pH 9.8 and c) pH 10.2. The dotted line indicates the solubility product of the ACC phase formed at each pH (pH 9.0/9.4: proto-calcite ACC; pH 9.8/10.2: proto-vaterite ACC).[27] The experiments showed the same trends as for PAsp and PGlu (Supplementary Figure 33), with increasing inhibition at decreasing pH values and the stabilization of a phase with higher solubility at high pH values. d) Quantitative evaluation of scale factors shows a stronger inhibition compared to PAsp/PGlu (see Figure 1a in the main text). At pH 9.0, no drop was detected in the titration experiments. Experimental details for titration experiments are described in the methods section in the main manuscript. Error bars represent ± 1-σ-standard deviation.

As described in the main manuscript, the most interesting effect is the strong nucleation inhibition of the polymers ("scale factor"), that also shows a strong pH dependency (Figure 1a in the main manuscript). It needs to be mentioned that there is also an increase in nucleation time in absence of additives if the pH value is lowered, as less carbonate is present in the carbonate-bicarbonate buffer equilibrium at a low pH values (4.4% CO_3^{2-} at pH 9.0 compared to 42.5% CO_3^{2-} at pH 10.2)[27] and more calcium needs to be added to reach a critical supersaturation. However, scale factors are reported relative to the reference experiment at the same pH value, and a clearly over-proportional increase in inhibition efficiency is detected

for low pH values. The strong scale inhibition by polymers was previously attributed to the (colloidal) stabilization of liquid precursor phases, visible by the growth of precursor droplets.[35,36] Indeed, this effect is also visible in our experiments, with the solution getting turbid before the maximum of free ion product is reached, indicating the formation of precursor droplets of a size detectable by the optrode (Supplementary Figure 35). Again, the early decrease in transmission is stronger at lower pH values (Supplementary Figure 35), indicating that the increasing efficiency of scale inhibition at lower pH values is linked to the stabilization of the dense liquid precursor phase, and that elucidating the mechanism of this stabilization is key to understanding the extraordinary crystallization inhibition properties of polycarboxylates.

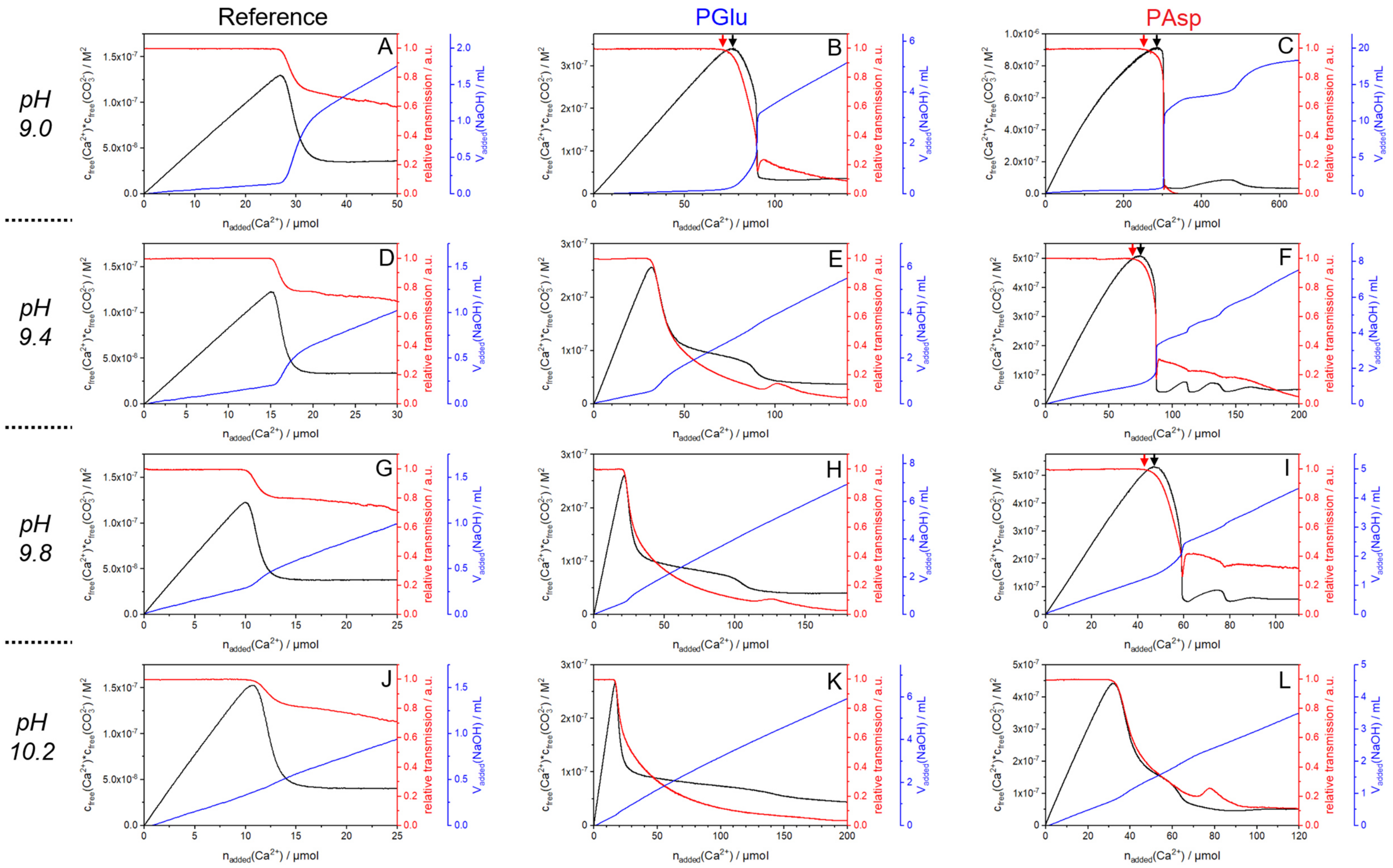

Supplementary Figure 35. Additional data for titration experiments. The pH dependent titration experiment for the polymer-free reference, PGlu and PAsp (10 mg/L polymer concentrations) are shown at a-c) pH 9.0, d-f) pH 9.4, g-i) pH 9.8 and j-l) pH 10.2. Experiments showed good repeatability (shown in Supplementary Figure 33). In addition to free ion product (black), the development of transmission of the solution detected by the optrode (red) and the amount of added NaOH to keep the pH constant (blue) is shown. For polymer experiments at lower pH values, a difference between drop in transmission (red arrow) and maximum in free ion product (black arrow) is visible, indicating the stabilization of liquid precursor phases by the polymers.[36]

4. Determination of single particle conductivity using C-AFM

4.1. Experimental strategy

To measure particle conductivity by C-AFM, the sample (usually stored as dispersion in acetone) was spin-coated on a gold coated Si wafer, that was then placed on the AFM holder and fixed using Ag conductive paint (Supplementary Figure 36a), as described in the methods section in the main manuscript.

In most works, nanoelectrodes are used to determine singly particle conductivity, with the particles being firmly connected to the electrodes.[37-39] In C-AFM however, the particles are not connected to the substrate and several difficulties can arise during conductivity measurement. For example, it was observed that during recording of a "conductivity map" in contact mode, the particles were pushed to the side by the AFM tip instead of being measured. We therefore decided to use non-contact mode (NCM) to look for interesting sample details and perform the conductivity measurements in the "spectroscopy mode", i.e., the tip is moved to the desired spot and then approached to press on the particle, followed by recording a current/voltage (I/V) diagram of this point. Using this strategy, the main difficulty is the apparent blindness during the measurement, as there is a time difference between recording the NCM AFM image and the measurement of the I/V diagram. It is therefore difficult to know, if a certain sample detail was actually hit by the AFM tip. This is especially challenging as we are interested in recording very small (<100 nm) particles. Combined with the particles lying loose on the wafer, the following problems could occur:

- The tip pushes the particle aside and the substrate is measured instead.
- The stage has a slow drift and until the spectroscopy measurement is started the particle has moved to a different position, causing it to not be measured.
- The particle is measured but sticks to the tip after measurement (tip poisoning), thereby introducing an error in the following measurements.
- The tip is hitting the particle but during the measurement the particle is lost, e.g., electrostatic interactions cause the particle change position or the strong current causes damage or decomposition of the particle.

To solve these issues, we have recorded the Z-height of the tip during the whole spectroscopy measurement. In this way, it can be tracked whether a particle is hit and if the particle undergoes changes during the measurement. In the following, an example is presented to explain this process. First, a NCM image to an select an interesting sample detail is recorded (exemplary shown in Supplementary Figure 36b). Then, spectroscopy measurement is performed on points across the image (usually a 20 x 20 grid is selected, Supplementary Figure 36c). On every point, an I/V diagram is recorded while the Z-height of the tip is recorded in parallel. If the measurement was successful, there is no change in Z-height during measurement (Supplementary Figure 36d). However, as already visible in the NCM height image (Supplementary Figure 36b), the structure shown as example is prone to moving. This is also visible during the spectroscopy measurement, as the Z-height decreases around 10 nm during the measurement of a different point (Supplementary Figure 36e). We would therefore

regard the second measurement (Supplementary Figure 36e) as invalid. In addition, as many points on the wafer/substrate are measured, the actual height of the sample detail can be calculated by the Z-height-difference between measurements on the wafer and on the sample detail. Usually, we are interested in smaller particles than those shown in Supplementary Figure 6, therefore before and after a measurement on a particle the wafer is measured, allowing us to check for changes of the wafer conductivity to exclude potential tip poisoning.

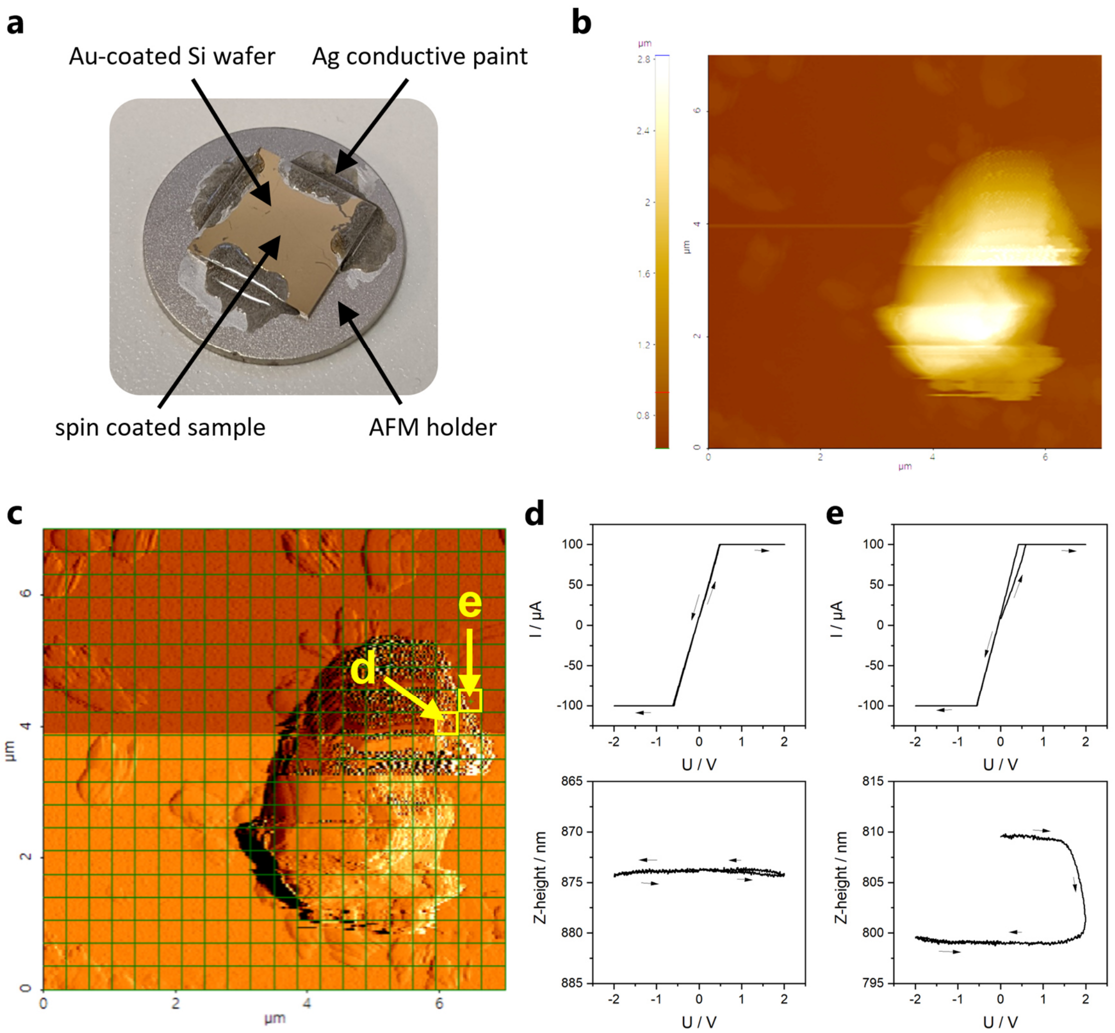

Supplementary Figure 36. Experimental determination of particle conductivity using C-AFM. A) Picture of the prepared sample on the AFM sample holder. B) Non-contact mode (NCM) height image of an agglomeration of gold nanoparticles. From the steps in the image, it is already evident that the particle moved during recording of the NCM image. C) NCM amplitude image with 20 x 20 grid for I/V spectroscopy measurements. Two points are highlighted, whose spectroscopy results are exemplary shown. D) Example for an I/V measurement (top) for a conductive particle. The maximum current was limited to ±100 µA. During the whole voltage screening, the Z-height (bottom) did not change, showing that no movement of the particle took place. E) I/V (top) and Z-height/V (bottom) diagrams for a point at which particle movement was detected. At the end of the measurement, the Z-height is 10 nm lower than at the beginning.

4.2. Evaluation of C-AFM data

In the following, evaluation of the data of C-AFM measurements is demonstrated for a sample of ACC nanoparticles. In the first step, AFM is performed in NCM to select a proper position for the C-AFM measurement, i.e., significant amounts of sample are present in the mapped region. After a detailed NCM map is recorded, the measurement mode is changed to C-AFM spectroscopy mode, and the spots for measuring the I/V diagrams are selected. Usually, a 20 x 20 grid is chosen for analysis (Supplementary Figure 37a) and 3 measurements across the grid are performed. After the data is recorded, the Z-height measured at the beginning and end of each measurement is analyzed, allowing to determine the "baseline" of Z-height of the wafer/substrate. Then, the spots at which a particle was hit, visible by an increase in Z-height, can be identified (Supplementary Figure 37b). The method allows to look at particles, or more precisely: Z-height offsets, down to an order of a few nm. Due to uneven surface of the wafer and the limited resolution of the AFM, the experimental Z-height measurement uncertainty is in the range of 2 nm. Therefore, data points with a Z-height difference of at least 2 nm relative to the wafer are selected for further analysis (Supplementary Figure 37c, marked points) while it is paid attention that the end Z-height is similar to the starting Z-height, i.e., that there was no movement of the particle during the measurement. Usually, the selected data points correspond well to the features visible in the NCM image (Supplementary Figure 37d), however, in some cases, there can be differences due to a small drift in the position of the wafer during the several hours of measurement time (e.g., point 84 in Supplementary Figure 37d: no particle is detected in NCM image, but spectroscopy measurement clearly shows an Z-height offset). In the last step, the I/V diagrams of the selected points are analyzed and the slope close to the origin of the graph (-0.2 V to 0.2 V) is determined. A higher slope of current vs. applied bias corresponds to a higher conductivity (Supplementary Figure 37e). For the final plots, this slope is plotted vs. the Z-height (relative to the level of the wafer) for each data point. In addition, points on the Au-coated substrate can be evaluated as a reference for the maximum detectably conductivity in the experiments.

As references, we performed measurements on conductive (Au) and non-conductive (vaterite) nanoparticles (Supplementary Figure 21), confirming that it is possible to determine conductivity of nanoparticles down to a size of 20 nm by this method. Below 20 nm, the conductivity seems to increase for all samples, probably due to leaking voltage, demonstrating the limits for this method (Supplementary Figures 23c and 24a). However, above a Z-height difference of 20 nm, the method can reliably qualitatively determine the conductivity of nanoparticles. Due to the large amounts of data points analyzed in each measurement, the method has great potential to analyze conductivity of nanoparticles with better statistics compared to other available methods.

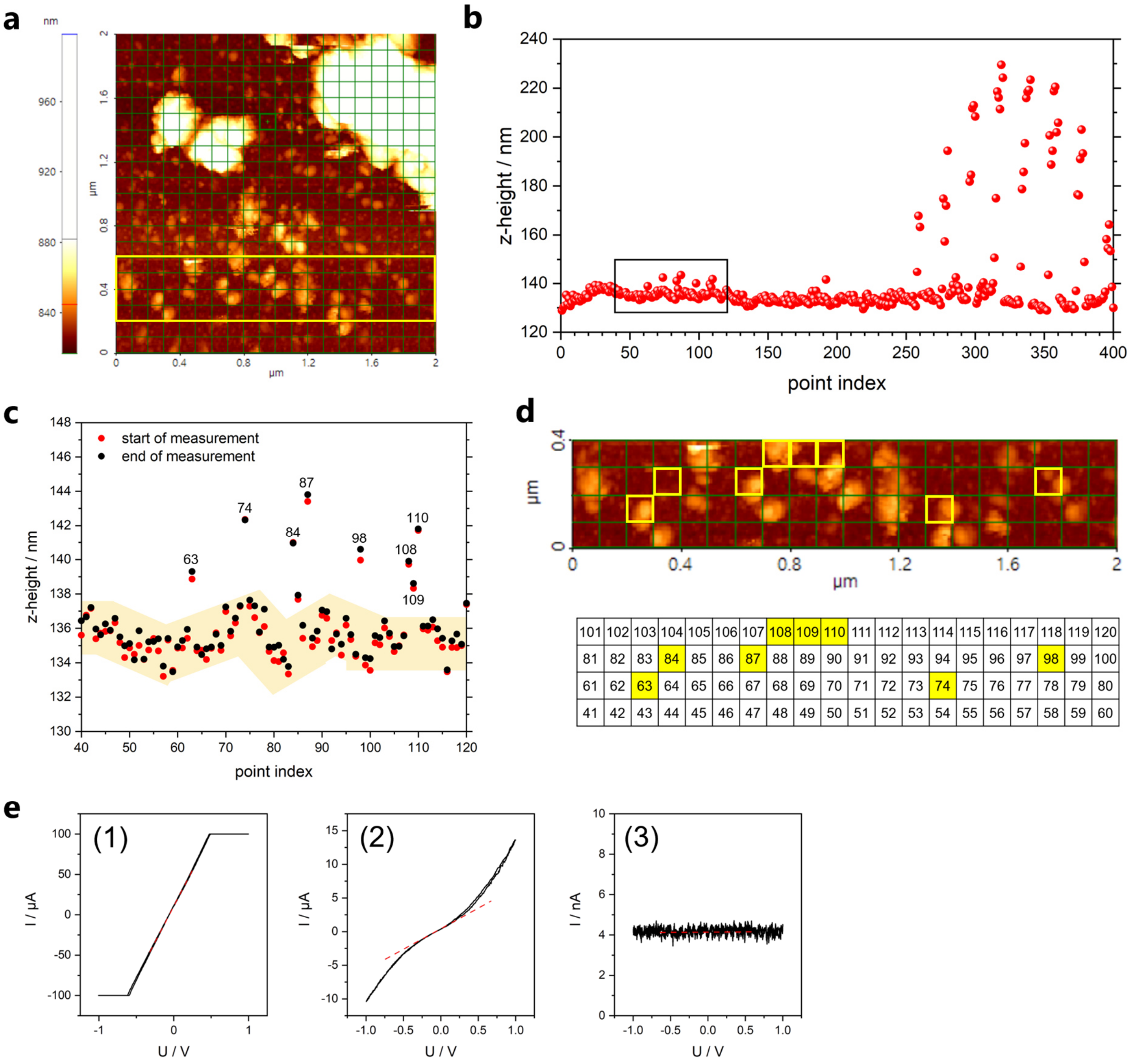

Supplementary Figure 37. Evaluation of C-AFM measurements. a) Non-contact mode (NCM) height map for a spin coated sample of ACC particles. Overlayed on the height map the 20 x 20 grid is shown, indicating the points at which C-AFM spectroscopy measurements were performed. The region highlighted in yellow (point 40 to 120) was used to demonstrate data evaluation. b) Starting Z-height of the I/V measurements for each data point. The area highlighted in black corresponds to the area highlighted in (a). The larger particles visible in the top part of the height map in (a) are also visible by the large Z-height starting from data point 250. c) Zoom in the region from point 40 to 120. Data points with a difference larger than 2 nm from the measurements on the wafer (yellow area at a Z-height of ~ 136 nm) were used for further analysis, provided the Z-height did not change during measurement. The labeled points were selected for further analysis. d) Illustration of the correspondence of selected data points on the grid (bottom, yellow highlighted) to the position on the AFM height image (top, yellow highlighted). Not all particles visible in the NCM image were hit during measurement, e.g., point 81, resulting in less points selected for evaluation as visible in the height map. In addition, not every selected point corresponds to a visible particle in the height image, e.g., point 84, showing that the wafer might have shifted between acquisition of the NCM image and C-AFM spectra. e) I/V diagrams for a particle showing (1) good, (2) moderate and (3) no conductivity. The slope of the diagram was used for qualitative comparison of samples.

Supplementary References

1 Platzer, G., Okon, M. & McIntosh, L. P. pH-dependent random coil ^{1}H, ^{13}C, and ^{15}N chemical shifts of the ionizable amino acids: a guide for protein pK_a measurements. *J. Biomol. NMR* **60**, 109-129 (2014).

2 Rabenstein, D. L. & Sayer, T. L. Carbon-13 chemical shift parameters for amines, carboxylic acids, and amino acids. *Journal of Magnetic Resonance (1969)* **24**, 27-39 (1976).

3 Khouzani, M. F. *et al.* Disordered amorphous calcium carbonate from direct precipitation. *CrystEngComm* **17**, 4842-4849 (2015).

4 Michel, F. M. *et al.* Structural characteristics of synthetic amorphous calcium carbonate. *Chem. Mater.* **20**, 4720-4728 (2008).

5 Nebel, H., Neumann, M., Mayer, C. & Epple, M. On the structure of amorphous calcium carbonate−A detailed study by solid-state NMR spectroscopy. *Inorg. Chem.* **47**, 7874-7879 (2008).

6 Ihli, J. *et al.* Dehydration and crystallization of amorphous calcium carbonate in solution and in air. *Nat. Commun.* **5**, 3169 (2014).

7 Heda, P. K. *et al.* A method of assessing solid state reactivity illustrated by thermal decomposition experiments on sodium bicarbonate. *Thermochim. Acta* **255**, 255-272 (1995).

8 Gebauer, D. *et al.* Proto-Calcite and Proto-Vaterite in Amorphous Calcium Carbonates. *Angew. Chem. Int. Ed.* **49**, 8889-8891 (2010).

9 Tudorachi, N. & Chiriac, A. P. TGA/FTIR/MS study on thermal decomposition of poly(succinimide) and sodium poly(aspartate). *Polym. Test.* **30**, 397-407 (2011).

10 McNeill, I. & Sadeghi, S. Thermal stability and degradation mechanisms of poly(acrylic acid) and its salts: Part 3 - Magnesium and calcium salts. *Polym. Degrad. Stab.* **30**, 267-282 (1990).

11 Farhadi-Khouzani, M., Chevrier, D. M., Zhang, P., Hedin, N. & Gebauer, D. Water as the key to proto-aragonite amorphous $CaCO_3$. *Angew. Chem. Int. Ed.* **55**, 8117-8120 (2016).

12 Swanson, H. E. & Tatge, E. Standard X-ray Diffraction Powder Patterns. *Natl. Bur. Stand. (U.S.)* **Circ. 539**, 58 (1953).

13 McMurdie, H. F. *et al.* Standard X-Ray Diffraction Powder Patterns from The JCPDS Research Associateship. *Powder Diffr.* **1**, 265-275 (1986).

14 Hogben, H. J., Krzystyniak, M., Charnock, G. T., Hore, P. J. & Kuprov, I. Spinach–a software library for simulation of spin dynamics in large spin systems. *Journal of Magnetic Resonance* **208**, 179-194 (2011).

15 Huang, Y.-C. *et al.* Uncovering the Role of Bicarbonate in Calcium Carbonate Formation at Near-Neutral pH. *Angew. Chem. Int. Ed.* **60**, 16707-16713 (2021).

16 Clark, S. J. *et al.* First principles methods using CASTEP. *Zeitschrift für kristallographie-crystalline materials* **220**, 567-570 (2005).

17 Kuprov, I. Fokker-Planck formalism in magnetic resonance simulations. *Journal of magnetic resonance* **270**, 124-135 (2016).

18 Freed, J. H., Bruno, G. V. & Polnaszek, C. F. Electron spin resonance line shapes and saturation in the slow motional region. *The Journal of Physical Chemistry* **75**, 3385-3399 (1971).

19 Sen, S., Kaseman, D. C., Colas, B., Jacob, D. E. & Clark, S. M. Hydrogen bonding induced distortion of CO_3 units and kinetic stabilization of amorphous calcium carbonate: Results from 2D ^{13}C NMR spectroscopy. *PCCP* **18**, 20330-20337 (2016).

20 Ramnarain, V. *et al.* Monitoring of $CaCO_3$ Nanoscale Structuration through Real-Time Liquid Phase Transmission Electron Microscopy and Hyperpolarized NMR. *J. Am. Chem. Soc.* (2022).

21 Gindele, M. B., Steingrube, L. V. & Gebauer, D. Generality of liquid precursor phases in gas diffusion-based calcium carbonate synthesis. *CrystEngComm* **23**, 7938-7943 (2021).

22 Wolf, S. L., Caballero, L., Melo, F. & Cölfen, H. Gel-like calcium carbonate precursors observed by in situ AFM. *Langmuir* **33**, 158-163 (2016).

23 Aizenberg, J., Muller, D. A., Grazul, J. L. & Hamann, D. Direct fabrication of large micropatterned single crystals. *Science* **299**, 1205-1208 (2003).
24 Taylor, G. F. The occurrence of monohydrocalcite in two small lakes in the south-east of South Australia. *American Mineralogist: Journal of Earth and Planetary Materials* **60**, 690-697 (1975).
25 Neumann, M. & Epple, M. Monohydrocalcite and Its Relationship to Hydrated Amorphous Calcium Carbonate in Biominerals. *Eur. J. Inorg. Chem.* **2007**, 1945-1945 (2007).
26 Kellermeier, M., Picker, A., Kempter, A., Cölfen, H. & Gebauer, D. A straightforward treatment of activity in aqueous $CaCO_3$ solutions and the consequences for nucleation theory. *Adv. Mater.* **26**, 752-757 (2014).
27 Gebauer, D., Völkel, A. & Cölfen, H. Stable prenucleation calcium carbonate clusters. *Science* **322**, 1819-1822 (2008).
28 Gindele, M. B., Malaszuk, K. K., Peter, C. & Gebauer, D. On the Binding Mechanisms of Calcium Ions to Polycarboxylates: Effects of Molecular Weight, Side Chain, and Backbone Chemistry. *Langmuir* **38**, 14409-14421 (2022).
29 Smeets, P. J. M. *et al.* A classical view on nonclassical nucleation. *Proc. Natl. Acad. Sci. U.S.A.* **114**, E7882-E7890 (2017).
30 Zou, Z., Bertinetti, L., Politi, Y., Fratzl, P. & Habraken, W. J. Control of Polymorph Selection in Amorphous Calcium Carbonate Crystallization by Poly(Aspartic Acid): Two Different Mechanisms. *Small* **13**, 1603100 (2017).
31 Njegić-Džakula, B., Falini, G., Brečević, L., Skoko, Ž. & Kralj, D. Effects of initial supersaturation on spontaneous precipitation of calcium carbonate in the presence of charged poly-L-amino acids. *J. Colloid Interface Sci.* **343**, 553-563 (2010).
32 Verch, A., Gebauer, D., Antonietti, M. & Cölfen, H. How to control the scaling of $CaCO_3$: A “fingerprinting technique” to classify additives. *Phys. Chem. Chem. Phys.* **13**, 16811-16820 (2011).
33 Sebastiani, F. *et al.* Water dynamics from THz spectroscopy reveal the locus of a liquid–liquid binodal limit in aqueous $CaCO_3$ solutions. *Angew. Chem. Int. Ed.* **56**, 490-495 (2017).
34 Bissi, V., Sun, C.-Y., Falini, G., Gilbert, P. U. & Mass, T. Coral acid rich protein selects vaterite polymorph *in vitro*. *J. Struct. Biol.* **209**, 107431 (2020).
35 Gebauer, D., Cölfen, H., Verch, A. & Antonietti, M. The multiple roles of additives in $CaCO_3$ crystallization: A quantitative case study. *Adv. Mater.* **21**, 435-439 (2009).
36 Schodder, P. I. *et al.* Probing the effects of polymers on the early stages of calcium carbonate formation by stoichiometric co-titration. *PCCP* **24**, 9978 - 9989 (2022).
37 Sun, L., Wang, J. & Bonaccurso, E. Conductivity of individual particles measured by a microscopic four-point-probe method. *Scientific reports* **3**, 1-5 (2013).
38 Sun, T., Bernabini, C. & Morgan, H. Single-colloidal particle impedance spectroscopy: Complete equivalent circuit analysis of polyelectrolyte microcapsules. *Langmuir* **26**, 3821-3828 (2010).
39 Dayen, J. *et al.* Nanotrench for nano and microparticle electrical interconnects. *Nanotechnology* **21**, 335303 (2010).
